# Volatiles from three genome sequenced fungi from the genus *Aspergillus*

**DOI:** 10.3762/bjoc.14.77

**Published:** 2018-04-24

**Authors:** Jeroen S Dickschat, Ersin Celik, Nelson L Brock

**Affiliations:** 1 Kekulé-Institute of Organic Chemistry and Biochemistry, University of Bonn, Gerhard-Domagk-Straße 1, 53121 Bonn, Germany; 2 Institute of Organic Chemistry, TU Braunschweig, Hagenring 30, 38106 Braunschweig, Germany (former address)

**Keywords:** *Aspergillus*, GC–MS, genomics, terpenes, volatiles

## Abstract

The volatiles emitted by agar plate cultures of three genome sequenced fungal strains from the genus *Aspergillus* were analysed by GC–MS. All three strains produced terpenes for which a biosynthetic relationship is discussed. The obtained data were also correlated to genetic information about the encoded terpene synthases for each strain. Besides terpenes, a series of aromatic compounds and volatiles derived from fatty acid and branched amino acid metabolism were identified. Some of these compounds have not been described as fungal metabolites before. For the compound ethyl (*E*)-hept-4-enoate known from cantaloupe a structural revision to the Z stereoisomer is proposed. Ethyl (*Z*)-hept-4-enoate also occurs in *Aspergillus clavatus* and was identified by synthesis of an authentic standard.

## Introduction

Ascomycete fungi are a highly productive and biosynthetically exceptionally creative source of secondary metabolites from all classes of natural products. Many prominent compounds such as lovastatin from *Aspergillus terreus* [1] or the penicillin antibiotics from *Penicillium* [2] are used for human wellfare, whilst others including aflatoxin from *Aspergillus flavus* [3] or the amatoxins from the death cap (*Amanita phalloides*) [4] are extremely toxic for humans. Recently, also volatile secondary metabolites from fungi attracted considerable interest [5–6]. Volatiles not only contribute to the pleasant aroma of edible mushrooms such as the penny bun (*Boletus edulis*) [7], but can also inhibit the growth of other fungi [8] which likely contributes to the induction of systemic resistance in plants by *Trichoderma* [9]. Fungal volatiles can also act as self-inhibitors of fungal germination [10] or as attractants for insects involved in spore distribution [11]. Furthermore, volatiles can be used as taxonomic markers [12] and can serve as indicators for fungal toxin production, e.g., the fungal emission of the sesquiterpene hydrocarbon trichodiene points to the production of trichothecene mycotoxins [13].

*Aspergillus* is a well-described genus comprising several hundreds of known species. Some of these species are human pathogens, e.g., *Aspergillus fumigatus* can cause infections especially in immunocompromised patients, while other species are safe, e.g., *Aspergillus oryzae* is traditionally used in Japanese sake brewing. The genus has a rich secondary metabolism with 807 compounds from 675 species that were recently summarised in the *Aspergillus* secondary metabolome database [14]. However, only a few studies about volatile natural products from *Aspergillus* are available [15–22]. Here we report on the volatiles released by three genome sequenced strains of *Aspergillus fischeri*, *A. kawachii* and *A. clavatus* and a correlation of the obtained analytical data to genome information.

## Results and Discussion

**Figure 1:**
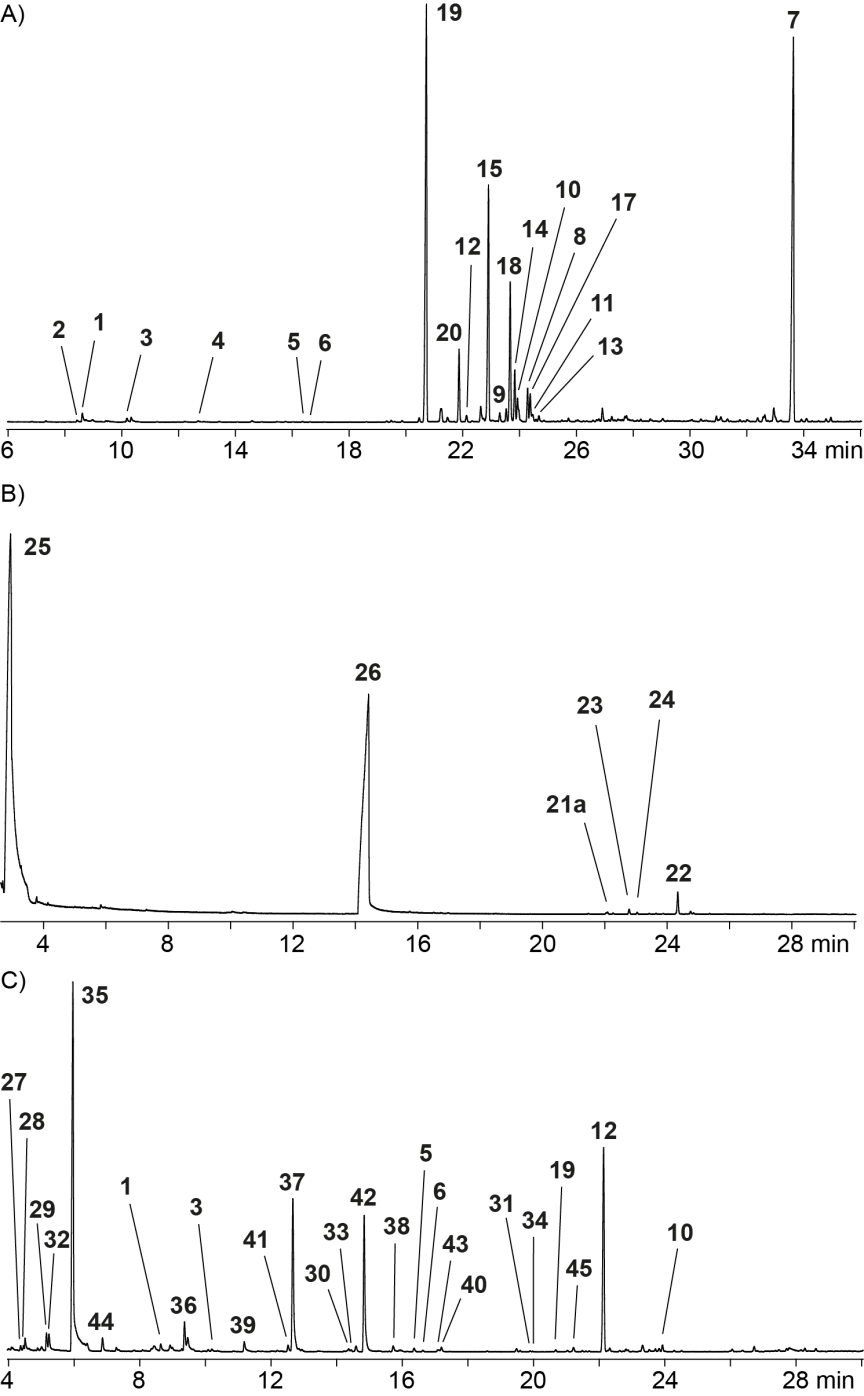
Total ion chromatograms of headspace extracts from A) *Aspergillus fischeri* NRRL 181, B) *Aspergillus kawachii* NBRC 4308, and C) *Aspergillus clavatus* NRRL 1. Numbers at peaks refer to compound numbers as defined in Table 1 and Schemes 1–5.

The volatiles released by agar plate cultures of *A. fischeri* NRRL 181, *A. kawachii* NBRC 4308 and *A. clavatus* NRRL 1 grown on medium 129 were collected on charcoal filters with a closed loop stripping apparatus (CLSA) [23]. After solvent extraction (CH_2_Cl_2_) of the filters the extracts were analysed by GC–MS and compounds were identified by comparison of the recorded EI mass spectra to mass spectral libraries and of calculated retention indices to literature data. For each investigated fungus a representative chromatogram of a headspace extract is shown in Figure 1.

### Aspergillus fisheri

**Scheme 1:**
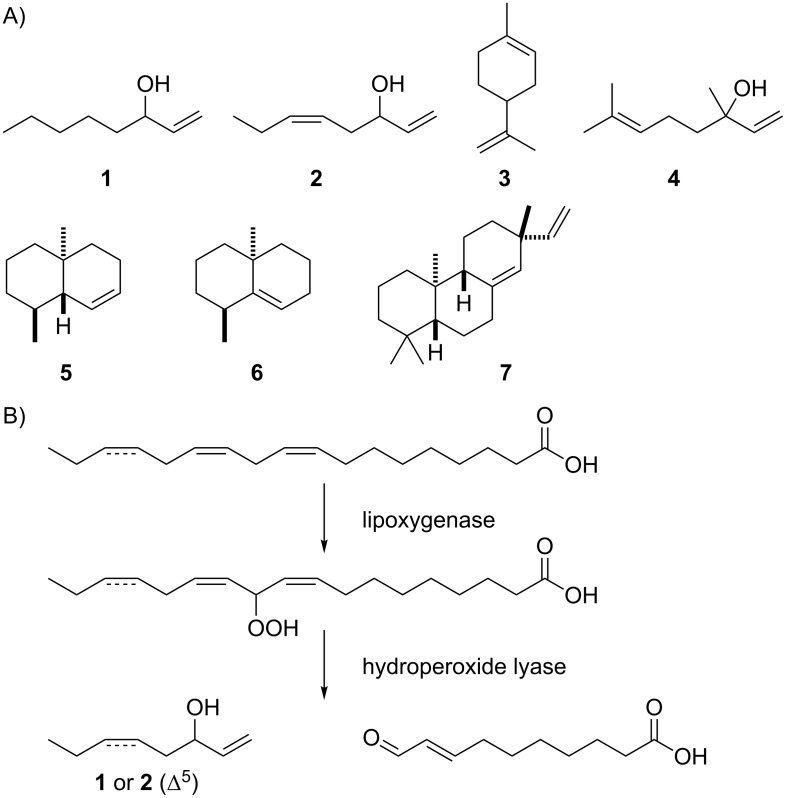
Volatiles from *Aspergillus fischeri*. For all chiral compounds in Schemes 1–5 the relative configurations are shown.

*A. fischeri* produced mainly terpenes, besides traces of the typical fungal volatile oct-1-en-3-ol (**1**) and a related compound that was tentatively identified as (*Z*)-octa-1,5-dien-3-ol (**2**) from its mass spectrum (Table 1 and Scheme 1A). Unfortunately, a retention index for **2** is not available from the literature, but the mass spectral database hit was very good and the assigned structure for **2** is biosynthetically reasonable: For compound **1** a biosynthetic pathway from linoleic acid via its hydroperoxide has been suggested [24–26], and if the same biosynthetic steps would proceed from linolenic acid, this would result in the assigned structure of **2** (Scheme 1B).

**Table 1:** Volatiles emitted by *Aspergillus fischeri* NRRL 181.

compound^a^	*I*^b^	*I* (lit.)^c^	ident.^d^	integral^e^

(*Z*)-octa-1,5-dien-3-ol (**2**)	974		ms (834)	0.1%
oct-1-en-3-ol (**1**)	979	974 [27]	ms (948), ri	0.5%
limonene (**3**)	1023	1024 [27]	ms (920), ri, std	0.2%
linalool (**4**)	1097	1095 [27]	ms (923), ri, std	0.1%
(8S*,9R*,10S*)-8,10-dimethyl-1-octalin (**5**)	1221	1224 [28]	ms (832), ri	<0.1%
(8S*,10R*)-8,10-dimethyl-1(9)-octalin (**6**)	1231	1233 [28]	ms (815), ri	<0.1%
daucene (**19**)	1378	1380 [27]	ms (917), ri	24.2%
*trans*-dauca-8,11-diene (**20**)	1424		ms (901)	3.4%
*trans*-α-bergamotene (**12**)	1434	1432 [27]	ms (898), ri	0.4%
α-acoradiene (**15**)	1465	1464 [27]	ms (937), ri	12.6%
*ar*-curcumene (**9**)	1482	1479 [27]	ms (882), ri	0.5%
isodaucene (**18**)	1496	1500 [27]	ms (918), ri	7.1%
cuparene (**14**)	1503	1504 [27]	ms (903), ri	2.5%
β-bisabolene (**10**)	1507	1505 [27]	ms (865), ri	1.7%
β-sesquiphellandrene (**8**)	1522	1521 [27]	ms (937), ri	1.6%
dauca-4(11),8-diene (**17**)	1526	1530 [27]	ms (964), ri	1.4%
(*E*)-γ-bisabolene (**11**)	1530	1529 [27]	ms (844), ri	0.5%
δ-cuprenene (**13**)	1539	1542 [27]	ms (818), ri	0.4%
pimara-8(14),15-diene (**7**)	1952	1948 [27]	ms (920), ri	25.6%

^a^Unidentified compounds and contaminants such as plasticisers are not listed. ^b^Retention index on a HP5-MS GC column. ^c^Retention index data from the literature. ^d^Compound identification is based on matching mass spectrum to a library spectrum (ms, match factor given in brackets, identical mass spectra would produce a match factor of 1000), identical or closely matching retention index (ri), comparison to an authentic standard (std). ^e^Percent of total peak area of the total ion chromatogram. The sum of integrals is lower than 100%, because unidentified compounds and contaminants are not included.

The other compounds identified in the headspace extracts of *A. fischeri* were all terpenes, including traces of the widespread monoterpenes limonene (**3**) and linalool (**4**). The C_12_ compounds (8S*,9R*,10S*)-8,10-dimethyl-1-octalin (**5**) and (8S*,10R*)-8,10-dimethyl-1(9)-octalin (**6**) are intermediates of the biosynthesis of the earthy odorant geosmin that is itself a degraded sesquiterpene [29–30], but geosmin could not be observed as a volatile of *A. fischeri*. The bacterial geosmin synthase is a class I terpene synthase (TS) with two domains [31] that occurs in many actinomycetes, cyanobacteria and myxobacteria, but fungal geosmin biosynthesis must require a different enzyme, because no homolog of the geosmin synthase is encoded in the genome of *A. fischeri* or of any other fungus. Furthermore, the diterpene pimara-8(14),15-diene (**7**) was one of the main compounds in the bouquet of *A. fischeri*. The biosynthesis of this compound is a two-step process that requires cyclisation of geranylgeranyl diphosphate (GGPP) to copalyl diphosphate (CPP) by a class II TS, followed by a second cyclisation event by a class I TS [32]. These reactions are likely catalysed by the only corresponding two-domain enzyme encoded in the *A. fischeri* genome (accession number XP_001264196, locus tag NFIA_009790). A phylogenetic analysis of 878 fungal terpene synthase homologs (Figure S1 in Supporting Information File 1) demonstrates that this enzyme is closely related to the bifunctional *ent*-copalyl diphosphate synthase/*ent*-kaurene synthase from *Fusarium fujikuroi* [33]. The N-terminal domain shows the DXDD motif that is typical for class II TSs (^312^DADD) and the C-terminal domain exhibits an aspartate-rich motif DDXXD, in this case with two of the usually found asparate residues exchanged by glutamate, and an NSE triad, a motif with highly conserved Asn, Ser and Glu residues, for Mg^2+^ binding as in class I TSs (^349^DEFME and ^847^NDYGSLARD).

**Scheme 2:**
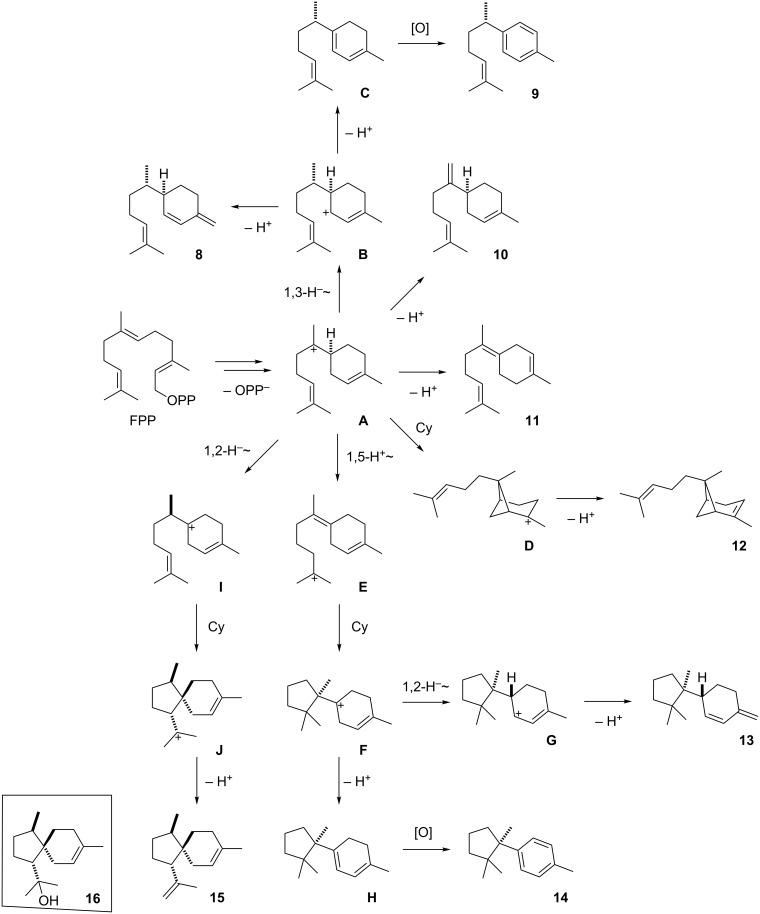
Biosynthesis of bisabolanes and related terpenes in *A. fischeri*.

Furthermore, two groups of structurally and biosynthetically related sesquiterpenes were found that could each arise from one sesquiterpene synthase (STS). The first of these groups comprised the main compound α-acoradiene (**15**), accompanied by minor amounts of β-sesquiphellandrene (**8**), *ar*-curcumene (**9**), β-bisabolene (**10**), (*E*)-γ-bisabolene (**11**), *trans*-α-bergamotene (**12**), δ-cuprenene (**13**), and cuparene (**14**). All these sesquiterpenes arise through a 1,6-cyclisation of farnesyl diphosphate (FPP, via nerolidyl diphosphate, NPP) to the bisabolyl cation (**A**, Scheme 2). A mixture of sesquiterpenes arising via cation **A** with the main product trichodiene was previously reported from *Fusarium* [34].

The compounds **10** and **11** are directly formed from this cation by deprotonation. A 1,3-hydride shift to **B** and deprotonation yields **8** and γ-curcumene (**C**). Instead of the latter compound its autoxidation product **9** is observed. From **A**, a second cyclisation event results in **D** that yields **12** upon deprotonation. Alternatively, **A** can react by a 1,5-proton shift to **E**, followed by cyclisation to **F** and deprotonation to **H**. This mechanism is favoured by quantum chemical calculations [35] and provides a reasonable alternative to a previously suggested cyclisation of **A** to a less stable secondary cation, followed by 1,4-hydride migration to yield the same intermediate **F** [36]. Again, the dihydrobenzene derivative **H** is not observed, but instead its autoxidation product **14** is detected. Finally, the main product **15** arises from **A** by a 1,2-hydride shift to the homobisabolyl cation **I**, cyclisation to **J**, and deprotonation.

**Scheme 3:**
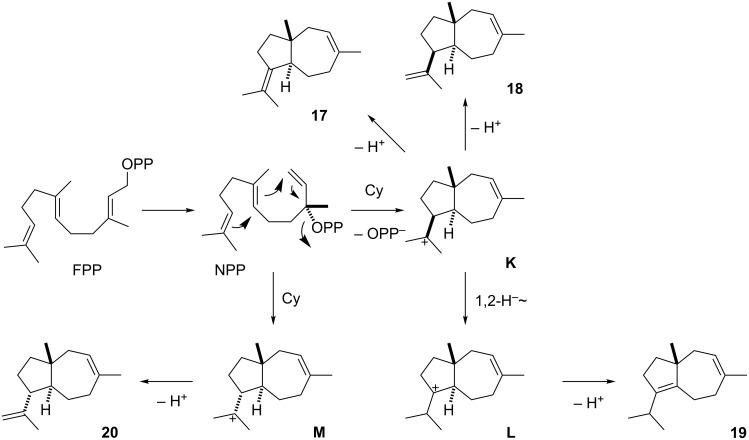
Biosynthesis of daucanes in *A. fischeri*.

The second group of biosynthetically related sesquiterpenes is composed of daucene (**19**), the main component in the headspace extracts from *A. fischeri*, and its congeners dauca-4(11),8-diene (**17**), isodaucene (**18**), and *trans*-dauca-8,11-diene (**20**). The biosynthesis of these compounds requires isomerisation of FPP to NPP, followed by cyclisation to **K** that results in **17** and **18** by deprotonation (Scheme 3). A 1,2-hydride shift to **L** and loss of a proton explains the main product **19**. For compound **20** a cyclisation of NPP with a different stereochemical course to **M** is required, followed by deprotonation. Two class I TSs are encoded in the genome of *A. fischeri* (accession numbers XP_001265719 and XP_001262485, locus tags NFIA_033880 and NFIA_030200), and it seems likely that each of these enzymes catalyses the formation of one of the two groups of sesquiterpenes with the main compounds α-acoradiene (**15**) and daucene (**19**). The enzyme XP_001262485 is closely related to the α-acorenol synthase from *Fusarium fujikuroi* [37] (Figure S1 in Supporting Information File 1) that produces α-acorenol (**16**) by quenching of cation **J** with water (box in Scheme 2), suggesting that this enzyme is responsible for the biosynthesis of **15** in *A. fischeri*. Therefore, the enzyme XP_001265719 is likely responsible for the biosynthesis of **19** and its byproducts. The biosynthetic origin of the observed traces of monoterpenes is unclear, but these compounds may be formed by a side activity of one of the TSs on GPP.

### Aspergillus kawachii

**Scheme 4:**
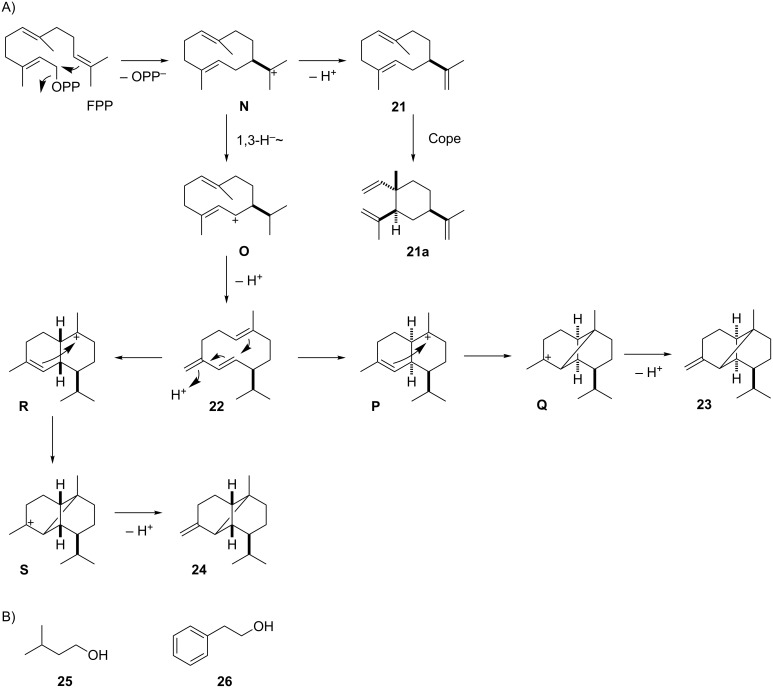
Volatiles from *A. kawachii*. A) Proposed biosynthesis of sesquiterpenes, B) other identified volatiles.

The bouquet of *A. kawachii* was dominated by the alcohols 3-methylbutan-1-ol (**25**) and 2-phenylethanol (**26**) that likely arise from leucine and phenylalanine metabolism, respectively (Figure 1, Table 2 and Scheme 4). In addition, small amounts of the sesquiterpenes β-elemene (**21a**), germacrene D (**22**), β-ylangene (**23**) and its stereoisomer β-copaene (**24**) were found. All these sesquiterpenes require a 1,10-cyclisation of FPP to the (*E*,*E*)-germacradienyl cation (**N**). Its deprotonation leads to germacrene A (**21**) that is known to undergo a Cope rearrangement to **21a** caused by the thermal impact during GC–MS analysis [38]. A 1,3-hydride shift transforms **N** into **O** that yields **22** by loss of a proton. Its reprotonation can induce a second cyclisation event via **R** and **S** to **24**, or with a different stereochemical course via **P** and **Q** to **23**. The mixture of **22**, **23** and **24**, accompanied by geosmin and the octalin **5**, has also been found in *Aspergillus niger*. Production of these compounds was shown to be upregulated in a knockout mutant of the MAP kinase Fus3 [39].

**Table 2:** Volatiles emitted by *Aspergillus kawachii* NBRC 4308.

compound^a^	*I*^b^	*I* (lit.)^c^	ident.^d^	integral^e^

3-methylbutan-1-ol (**25**)	<800	731 [27]	ms (916), std	61.2%
2-phenylethanol (**26**)	1110	1106 [27]	ms (927), ri, std	36.3%
β-elemene (**21a**)	1391	1389 [27]	ms (855), ri	0.1%
β-ylangene (**23**)	1419	1419 [27]	ms (901), ri	0.2%
β-copaene (**24**)	1430	1430 [27]	ms (903), ri	0.1%
germacrene D (**22**)	1483	1484 [27]	ms (934), ri	0.9%

^a^Unidentified compounds and contaminants such as plasticisers are not listed. ^b^Retention index on a HP5-MS GC column. ^c^Retention index data from the literature. ^d^Compound identification is based on matching mass spectrum to a library spectrum (ms, match factor given in brackets, identical mass spectra would produce a match factor of 1000), identical or closely matching retention index (ri), comparison to an authentic standard (std). ^e^Percent of total peak area of the total ion chromatogram. The sum of integrals is lower than 100%, because unidentified compounds and contaminants are not included.

The genome of *A. kawachii* contains three genes for TS homologs (accession numbers GAA83682, GAA88217 and GAA91251, locus tags AKAW_01797, AKAW_06331 and AKAW_09365). The first enzyme GAA83682 shows close homology to the bifunctional *ent*-kaurene synthases from *Fusarium* and is likely involved in diterpene biosynthesis. The fact that no corresponding diterpene was observed may point to a low gene expression under laboratory culture conditions. It is currently not possible to conclude which of the other two TSs are involved in the biosynthesis of the observed sesquiterpenes from *A. kawachii*. Notably, both enzymes GAA88217 and GAA91251 are closely related to fungal germacrene D synthases [40] (Figure S1 in Supporting Information File 1) and are good candidates for the formation of **22** and the compounds derived from it in *A. kawachi*.

### Aspergillus clavatus

**Scheme 5:**
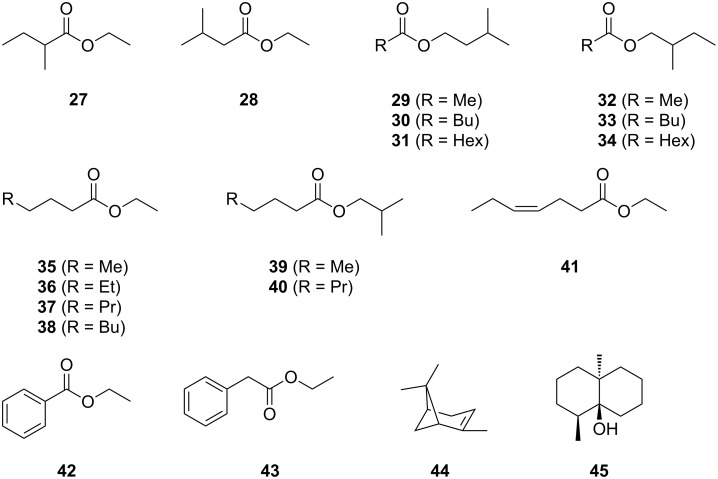
Volatiles from *A. clavatus*.

The headspace extracts from *A. clavatus* contained small amounts of oct-1-en-3-ol (**1**) and terpenes, including limonene (**3**) and pinene (**44**), and the octalins **5** and **6**, in this species accompanied by geosmin (**45**) (Figure 1, Table 3 and Scheme 5). Furthermore, daucene (**19**) was observed in small amounts, which further supports the functional assignment of the enzyme XP_001265719 from *A. fischeri* for the biosynthesis of this compound, because the phylogenetic analysis of fungal TSs (Figure S1 in Supporting Information File 1) reveals a closely homologous enzyme in *A. clavatus* (accession number XP_001273061, locus tag ACLA_093340). Only the gene expression level in laboratory cultures of *A. clavatus* seems to be much lower than for *A. fischeri*, since **19** was the main headspace constituent of *A. fischeri*, but only emitted in traces by *A. clavatus*. In addition, β-bisabolene (**10**) and *trans*-α-bergamotene (**12**) were released by *A. clavatus*. These compounds have also been observed in *A. fischeri* where they seem to be side products of α-acoradiene biosynthesis. At the current stage it remains elusive which of the three other TS homologs (accession numbers XP_001272213, XP_001273847 and XP_001273868, locus tags ACLA_052600, ACLA_063920 and ACLA_064130) in *A. clavatus* may catalyse the formation of **10** and **12**. The enzyme XP_001276070 (ACLA_076850), likely a bifunctional diterpene synthase (DTS), seems to be not expressed in laboratory culture, but another function cannot be excluded for this enzyme.

**Table 3:** Volatiles emitted by *Aspergillus clavatus* NRRL 1.

compound^a^	*I*^b^	*I* (lit.)^c^	ident.^d^	integral^e^

ethyl 2-methylbutyrate (**27**)	849	850 [41]	ms (911)	0.3%
ethyl 3-methylbutyrate (**28**)	853	849 [27]	ms (915), ri	0.3%
3-methylbutyl acetate (**29**)	879	869 [27]	ms (958), ri	1.0%
2-methylbutyl acetate (**32**)	883	875 [27]	ms (955), ri	1.4%
ethyl pentanoate (**35**)	908	901 [27]	ms (935), ri	33.0%
α-pinene (**44**)	932	932 [27]	ms (929), ri	1.0%
oct-1-en-3-ol (**1**)	979	974 [27]	ms (899), ri	0.7%
ethyl hexanoate (**36**)	1000	997 [27]	ms (955), ri	1.9%
limonene (**3**)	1024	1024 [27]	ms (857), ri	0.2%
isobutyl pentanoate (**39**)	1052		std	0.9%
ethyl (*Z*)-hept-4-enoate (**41**)	1092		std	0.4%
ethyl heptanoate (**37**)	1096	1097 [27]	ms (961), ri	11.4%
3-methylbutyl pentanoate (**30**)	1150	1152 [42]	std	0.3%
2-methylbutyl pentanoate (**33**)	1153		std	0.1%
ethyl benzoate (**42**)	1167	1169 [27]	ms (938), ri	9.9%
ethyl octanoate (**38**)	1194	1196 [27]	ms (896), ri	0.5%
(8S*,9R*,10S*)-8,10-dimethyl-1-octalin (**5**)	1221	1224 [28]	ms (868), ri	0.3%
(8S*,10R*)-8,10-dimethyl-1(9)-octalin (**6**)	1231	1233 [28]	ms (850), ri	0.1%
ethyl phenylacetate (**43**)	1242	1243 [27]	ms (867), ri	0.2%
isobutyl heptanoate (**40**)	1246		std	0.4%
3-methylbutyl heptanoate (**31**)	1343		std	<0.1%
2-methylbutyl heptanoate (**34**)	1347		std	<0.1%
daucene (**19**)	1378	1380 [27]	ms (851), ri	0.2%
geosmin (**45**)	1395	1399 [27]	ms (895), ri	0.4%
*trans*-α-bergamotene (**12**)	1434	1432 [27]	ms (964), ri	13.5%
β-bisabolene (**10**)	1507	1505 [27]	ms (926), ri	0.5%

^a^Unidentified compounds and contaminants such as plasticisers are not listed. ^b^Retention index on a HP5-MS GC column. ^c^Retention index data from the literature. ^d^Compound identification is based on matching mass spectrum to a library spectrum (ms, match factor given in brackets, identical mass spectra would produce a match factor of 1000), identical or closely matching retention index (ri), comparison to an authentic standard (std). ^e^Percent of total peak area of the total ion chromatogram. The sum of integrals is lower than 100%, because unidentified compounds and contaminants are not included.

Esters were the predominant class of compounds emitted by *A. clavatus*. The observed pattern of volatiles was very unusual, because many ethyl esters and esters derived from carboxylic acids with an odd number of carbons were found. Since the carboxylic acid portion usually derives from fatty acid biosynthesis, a process in which the C_2_ starter acetyl-CoA is elongated with C_2_ units, esters from carboxylic acids with an even number of carbons are much more widespread. Furthermore, esterification with *S*-adenosyl-l-methionine (SAM) by a methyltransferase is a very common process in nature, while ethyl esters are rarer and likely require a two-step pathway through reduction of acetyl-CoA to ethanol and its esterification by an acyl transferase. In *Neurospora crassa* acids of short chain alcohols are formed from alcohols and aldehydes via hemiacetals that are oxidised to the corresponding esters by an alcohol dehydrogenase [43].

The main compounds were ethyl pentanoate (**35**) and ethyl heptanoate (**37**), accompanied by small amounts of ethyl hexanoate (**36**), ethyl octanoate (**38**) and the unsaturated ester ethyl (*Z*)-hept-4-enoate (**41**) that was unambiguously identified by synthesis of a reference compound by esterification of (*Z*)-hept-4-enoic acid with ethanol. A compound with the retention index *I* = 1090 was reported from cantaloupe (*Curcumis melo*) and tentatively identified as ethyl (*E*)-hept-4-enoate [44], but the *E* stereoisomer should elute significantly later than the *Z* isomer. Likely, the reported compound is the same as found here and the structure requires correction to ethyl (*Z*)-hept-4-enoate. Further ethyl esters were ethyl 2-methylbutyrate (**27**) and ethyl 3-methylbutyrate (**28**), and the aromatic esters ethyl benzoate (**42**) and ethyl phenylacetate (**43**). The esters **27** and **28** were reported previously from an *Aspergillus parasiticus* knockout mutant of the global regulator VeA, but not from the wildtype [20], while **27** is known from *Schizophyllum commune* [45] and **28** was recently found in truffle [46]. The ester **42** has been described from *A. clavatus* before [16].

Besides ethyl esters, a series of esters derived from branched short chain alcohols was identified, including the widespread compounds [5] 3-methylbutyl acetate (**29**) and 2-methylbutyl acetate (**32**). The alcohol portion of these esters likely originates from leucine and isoleucine through transamination to the corresponding α-ketocarboxylic acid, oxidative decarboxylation and reduction. For the pentanoate and heptanoate esters not only the corresponding compounds 3- and 2-methylbutyl pentanoate (**30** and **33**) and -heptanoate (**31** and **34**), but also the valine-derived analogues isobutyl pentanoate (**39**) and -heptanoate (**40**) were detected. Since only for **30** a published retention index was available [42], all six esters were synthesised for their unambiguous identification. Compound **30** was previously tentatively identified from *Nodulisporium* [47], while the other esters of this series **31**, **33**, **34**, **39**, and **40** have not been reported from fungi before.

## Conclusion

In summary, the volatiles from three species of the genus *Aspergillus* were identified. *A. kawachii* released large amounts of 3-methylbutan-1-ol (**25**) and 2-phenylethanol (**26**), besides traces of germacrene D (**22**) and a few other terpenes derived from it. This organism encodes two TSs that are closely related to fungal germacrene D synthases that could both be involved in the biosynthesis of the observed sesquiterpenes. The volatiles profile of *A. fischeri* was dominated by the terpenes α-acoradiene (**15**), daucene (**19**) and pimara-8(14),15-diene (**7**) which matches the genome sequence information from this organism: there is one encoded bifunctional DTS likely for the biosynthesis of **7**, and two STSs likely for **15** and **19**. One of these enzymes is similar to the α-acorenol synthase from *F. fujikuroi*, suggesting that this enzyme is responsible for the biosynthesis of **15**. The remaining STS can be assigned to **19** which is supported by the occurrence of a closely related enzyme in *A. clavatus* that also produces small amounts of **19**. The biosynthetic byproduct dauca-4(11),8-diene (**17**) has recently also been described from the sponge isolate *Dichotomomyces cejpii* [48], but the genome of this fungus is not sequenced and the presence of a closely related TS in this organism is currently unknown. While these analyses demonstrate that a reasonable correlation of data from chemical analyses to genomic data allows for a tentative assignment of functions to biosynthetic enzymes, this work cannot replace their necessary biochemical characterisation, but the approach presented here can help to identify interesting candidate enzymes for further investigation. Furthermore, this work demonstrates that fungal volatiles are an interesting subject of study, as many of the compounds such as several of the identified esters from *A. clavatus* have not been reported from fungi before.

## Supporting Information

File 1 Phylogenetic tree of fungal type I terpene synthases, experimental procedures and NMR spectra of synthetic compounds.
