# Supplementary material for: Volatiles from three genome sequenced fungi from the genus Aspergillus
Source: Beilstein J Org Chem. 2018 Apr 24;14:900–10. doi: 10.3762/bjoc.14.77 (PMC5942377; doi:10.3762/bjoc.14.77)
Supplement: File 1 — Phylogenetic tree of fungal type I terpene synthases, experimental procedures and NMR spectra of synthetic compounds. [file Beilstein_J_Org_Chem-14-900-s001.pdf]

Supporting Information

for

Volatiles from three genome sequenced fungi from the genus *Aspergillus*

Jeroen S. Dickschat*[1], Ersin Celik[1] and Nelson L. Brock[2]

Address: [1]Kekulé-Institute of Organic Chemistry and Biochemistry, University of Bonn, Gerhard-Domagk-Straße 1, 53121 Bonn, Germany and [2]Institute of Organic Chemistry, TU Braunschweig, Hagenring 30, 38106 Braunschweig, Germany (former address)

Email: Jeroen S. Dickschat - dickschat@uni-bonn.de

* Corresponding author

Phylogenetic tree of fungal type I terpene synthases, experimental procedures and NMR spectra of synthetic compounds

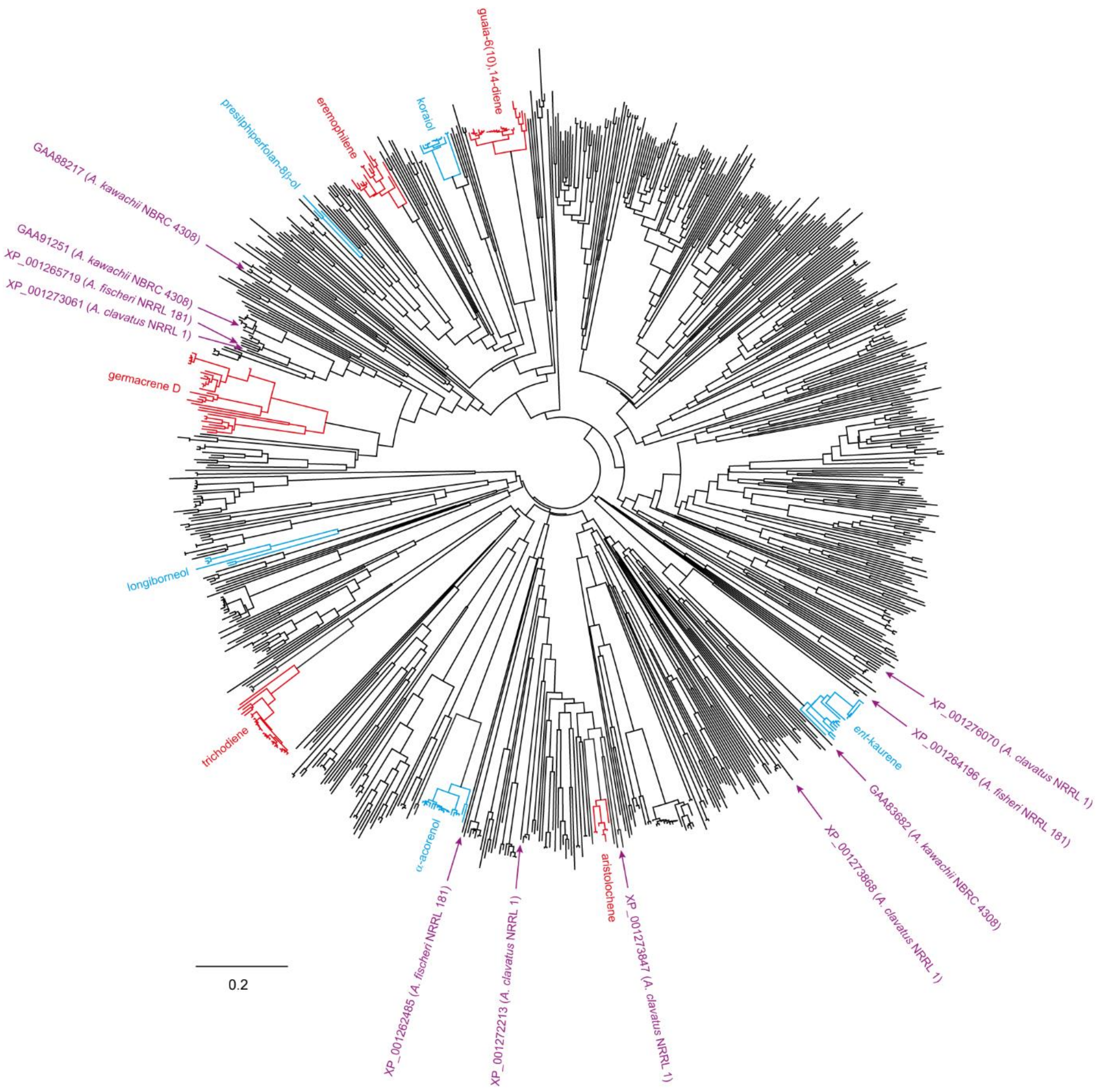

Figure S1: Phylogenetic tree of fungal type I terpene synthases. Groups of closely related enzymes that likely make the same product are shown in red and blue, terpene synthases encoded in the genomes of the three investigates species of Aspergillus are shown by purple arrows.

Experimental

Strains and culture conditions

Aspergillus clavatus NRRL 1 and *Aspergillus fischeri* NRRL 181 were obtained from the USDA strain collection, *Aspergillus kawachii* NBRC 4308 was obtained from the NITE Biological Resource Center. All strains were grown on medium 129 agar plates (1 L infusion from potatoes, 20 g glucose, 15 g agar). For the preparation of infusion from potatoes, scrubbed and sliced potatoes (200 g) are boiled in water for 1 h. The infusion is passed through a fine sieve and directly used for medium preparation.

Preparation of headspace extracts

After inoculation with the different *Aspergillus* strains, agar plates were incubated at 28 °C for 3–4 days until plates were fully grown. The plates were placed in a self-made closed-loop stripping apparatus as described by Grob and Zürcher [1] that directs a circulating air stream over the agar plate and then through a charcoal filter (precision charcoal filter BB91006015, Chromtech, Idstein, Germany). After a collection time of 24 h the filter was removed and extracted with CH_2Cl_2 (50 μL), followed by direct GC–MS analysis of the obtained extracts.

GC–MS

For GC–MS analyses a system of a 7890B GC connected to a 5977A mass detector (Agilent) was used. The GC was equipped with a HP5-MS fused silica capillary column (30 m, 0.25 mm i. d., 0.50 μm film). GC instrumental settings were 1) temperature program: 5 min at 50 °C increasing at 5 °C min^{-1} to 320 °C, 2) injection volume: 2 μL, 3) split ratio: 10:1, 60 s valve time, and 4) carrier gas: He at 1 mL

min^{-1}. MS instrumental settings were 1) inlet pressure: 77.1 kPa, He at 23.3 mL min^{-1}, 2) transfer line: 250 °C, and 3) electron energy: 70 eV. Retention indices (*I*) were determined from a homologous series of *n*-alkanes (C_7–C_{40}).

Synthesis of esters 30, 31, 33, 34, 39, 40 and 41

The esters were synthesised either from acyl chlorides using a published method A [2] or from carboxylic acids through published method B [3].

3-Methylbutyl pentanoate (**30**). Method B, starting material: 2.04 g (20.0 mmol), yield: 2.78 g (16.2 mmol, 81%). GC (HP-5): *I* = 1150; ^{1}H NMR (500 MHz, $CDCl_3$): δ = 4.09 (t, *J* = 6.9 Hz, 2H), 2.29 (dd, *J* = 7.9, 7.2 Hz, 2H), 1.68 (dt, *J* = 13.5, 6.7 Hz, 1H), 1.64 – 1.56 (m, 2H), 1.51 (q, *J* = 6.9 Hz, 2H), 1.39 – 1.30 (m, 2H), 0.95 – 0.87 (m, 9H) ppm; ^{13}C NMR (125 MHz, $CDCl_3$): δ = 174.1 (C_q), 63.1 (CH_2), 37.5 (CH_2), 34.3 (CH_2), 27.2 (CH_2), 25.2 (CH), 22.6 (2xCH_3), 22.4 (CH_2), 13.9 (CH_3) ppm; EI-MS (70 eV): *m*/*z* (%) = 129 (4), 85 (61), 70 (100), 55 (27), 41 (17).

3-Methylbutyl heptanoate (**31**). Method A, starting material: 4.46 g (30.0 mmol, yield: 4.92 g (24.6 mmol, 82%). GC (HP-5): *I* = 1343; ^{1}H NMR (500 MHz, $CDCl_3$): δ = 4.09 (t, *J* = 6.9 Hz, 2H), 2.32 – 2.24 (m, 2H), 1.69 (dq, *J* = 13.5, 6.7 Hz, 1H), 1.65 – 1.57 (m, 2H), 1.51 (q, *J* = 6.9 Hz, 2H), 1.35 – 1.25 (m, 6H), 0.92 (d, *J* = 6.7 Hz, 6H), 0.90 – 0.84 (m, 3H) ppm; ^{13}C NMR (125 MHz, $CDCl_3$): δ = 174.2 (C_q), 63.0 (CH_2), 37.5 (CH_2), 34.6 (CH_2), 31.6 (CH_2), 29.0 (CH_2), 25.2 (CH), 25.1 (CH_2), 22.6 (CH_2), 22.6 (2xCH_3), 14.2 (CH_3) ppm; EI-MS (70 eV): *m*/*z* (%) = 131 (14), 113 (36), 70 (100), 55 (19), 43 (33).

2-Methylbutyl pentanoate (**33**). Method A, starting material: 2.04 g, (20.0 mmol), yield: 2.69 g (15.6 mmol, 78%). GC (HP-5): *I* = 1153; ^{1}H NMR (300 MHz $CDCl_3$): δ = 3.98 (dd, *J* = 10.9, 6.1, 1H), 3.88 (dd, *J* = 10.8, 6.8, 1H), 2.31 (t, *J* = 7.5 Hz, 2H), 1.78 – 1.55 (m, 3H), 1.52 – 1.28 (m, 3H), 1.26 – 1.08 (m, 1H), 0.97 – 0.82 (m, 9H) ppm.

^{13}C NMR (75 MHz $CDCl_3$): δ = 174.2 (C_q), 69.1 (CH_2), 34.3 (CH_2), 34.3 (CH), 27.3 (CH_2), 26.2 (CH_2), 22.4 (CH_2), 16.6 (CH_3), 13.9 (CH_3), 11.4 (CH_3) ppm; EI-MS (70 eV): m/Z (%) = 116 (4), 85 (100), 70 (60), 57 (31),43 (13).

2-Methylbutyl heptanoate (**34**). Method A, starting material: 4.46 g (30.0 mmol); yield: 4.98 g (24.9 mmol, 83%). GC (HP-5): *I* = 1347; ^{1}H NMR (500 MHz, $CDCl_3$): δ = 3.95 (dd, *J* = 10.7, 6.0 Hz, 1H), 3.86 (dd, *J* = 10.8, 6.7 Hz, 1H), 2.30 (t, *J* = 7.5 Hz, 2H), 1.70 (m, 1H), 1.62 (m, 2H), 1.43 (m, 1H), 1.36 – 1.24 (m, 6H), 1.18 (m, 1H), 0.97 – 0.78 (m, 9H) ppm; ^{13}C NMR (125 MHz, $CDCl_3$): δ = 174.2 (C_q), 69.0 (CH_2), 34.6 (CH_2), 34.3 (CH), 31.6 (CH_2), 29.0 (CH_2), 26.2 (CH_2), 25.2 (CH_2), 22.6 (CH_2), 16.5 (CH_3), 14.2 (CH_3), 11.4 (CH_3) ppm; EI-MS (70 eV): *m*/*z* (%) = 131 (13), 113 (100), 70 (86), 57 (14), 43 (32).

Isobutyl pentanoate (**39**). Method B, starting material: 2.04 g (20.0 mmol), yield: 2.40 g (15.2 mmol, 76%). GC (HP-5): *I* = 1052; ^{1}H NMR (500 MHz, $CDCl_3$): δ = 3.85 (d, *J* = 6.7 Hz, 2H), 2.31 (t, *J* = 7.5, 2H), 1.92 (nonet, *J* = 6.7 Hz, 1H), 1.61 (quintet, *J* = 7.6 Hz, 2H), 1.36 (sextet, *J* = 7.4 Hz, 2H), 0.96 – 0.84 (m, 9H) ppm; ^{13}C NMR (125 MHz, $CDCl_3$): δ = 174.1 (C_q), 70.5 (CH_2), 34.3 (CH_2), 27.9 (CH), 27.3 (CH_2), 22.4 (CH_2), 19.3 (2xCH_3), 13.9 (CH_3) ppm. EI-MS (70 eV): *m*/*z* (%) = 115 (9), 103 (24), 85 (100), 57 (58), 41 (22).

Isobutyl heptanoate (**40**). Method A, starting material: 4.46 g (30.0 mmol), yield: 5.08 g (27.3 mmol, 91%). GC (HP-5): *I* = 1246; ^{1}H NMR (500 MHz, $CDCl_3$): δ = 3.85 (d, *J* = 6.7 Hz, 2H), 2.31 (t, *J* = 7.5 Hz, 2H), 1.92 (nonet, *J* = 6.7 Hz, 1H), 1.62 (m, 2H), 1.36 – 1.25 (m, 8H), 0.97 – 0.83 (m, 9H) ppm; ^{13}C NMR (125 MHz, $CDCl_3$): δ = 174.1 (C_q), 70.5 (CH_2), 34.6 (CH_2), 31.6 (CH_2), 29.0 (CH_2), 27.9 (CH), 25.2 (CH_2), 22.6 (CH_2), 19.3 (2xCH_3), 14.2 (CH_3) ppm; EI-MS (70 eV): *m*/*z* (%) = 131 (37), 113 (100), 85 (14), 56 (50), 43 (21).

Ethyl (*Z*)-hept-4-enoate (**41**). Method B, starting material: 0.26 g (2.0 mmol), yield: 0.26 g (1.64 mmol, 82%). GC (HP-5): *I* = 1092; ^{1}H NMR (500 MHz, $CDCl_3$): δ = 5.46 – 5.37 (m, 1H), 5.33 – 5.27 (m, 1H), 4.13 (q, *J* = 7.1 Hz, 2H), 2.42 – 2.28 (m, 4H), 2.13 – 2.01 (m, 2H), 1.25 (t, *J* = 7.1 Hz, 3H), 0.96 (t, *J* = 7.5 Hz, 3H) ppm; ^{13}C NMR (125 MHz, $CDCl_3$): δ = 173.4 (C_q), 133.3 (=CH), 127.0 (=CH), 60.4 (CH_2), 34.6 (CH_2), 22.9 (CH_2), 20.6 (CH_2), 14.4 (2xCH_3) ppm; EI-MS (70 eV): *m*/*z* (%) = 156 (35), 110 (100), 88 (86), 68 (80), 55 (55), 41 (46).

References

1. Grob, K.; Zürcher, F.; *J. Chromatogr. A,* **1976,** *117,* 285–294.

2. Strohalm, H.; Dregus, M.; Wahl, A.; Engel, K. H. *J. Agric. Food Chem.,* **2007,** *55,* 10339–10344.

3. Moumne, R.; Lavielle, S.; Karoyan, P. *J. Org. Chem.,* **2006,** *71,* 3332–3334.

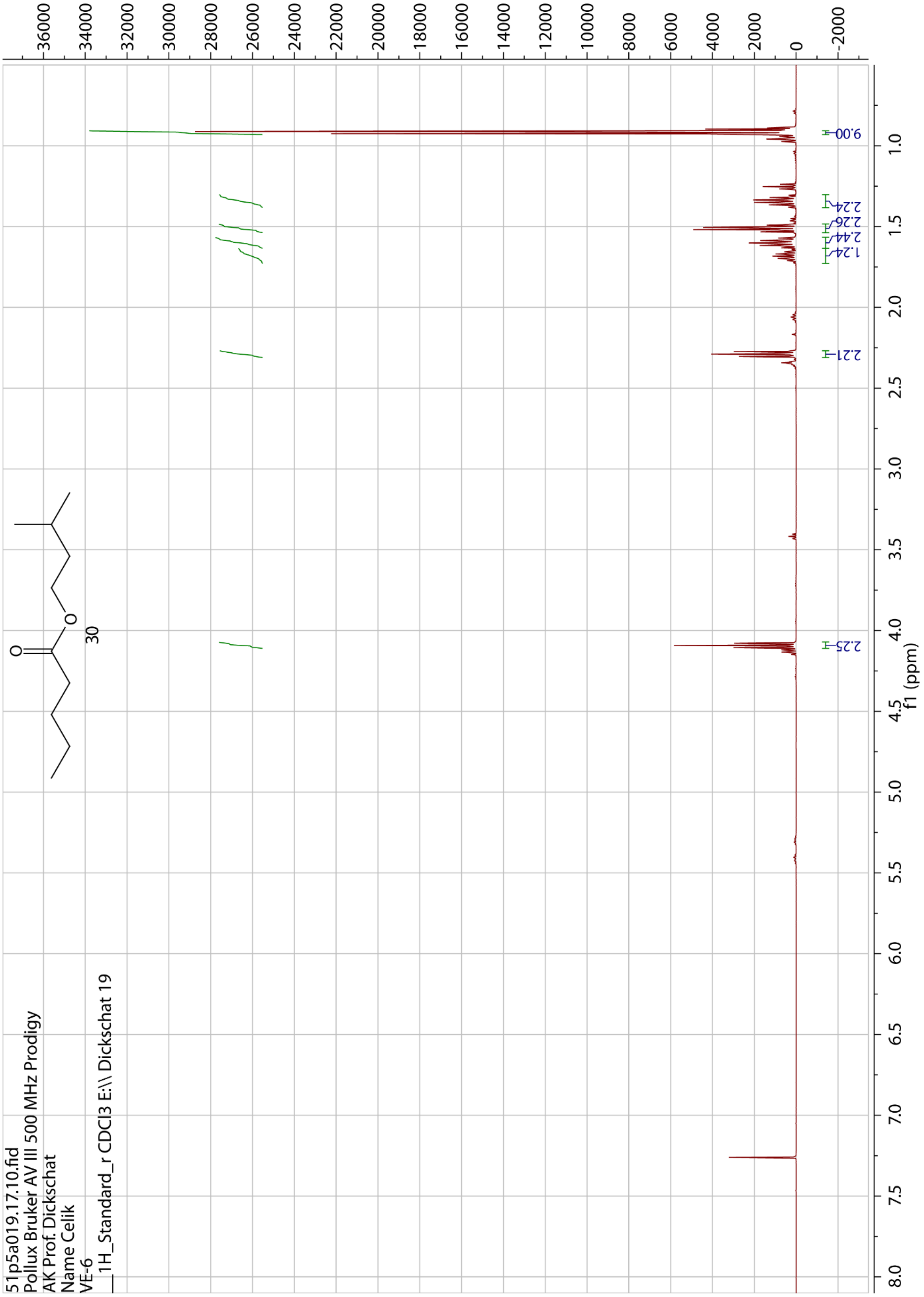

Figure S2: ^{1}H NMR spectrum of **30** (500 MHz, $CDCl_3$).

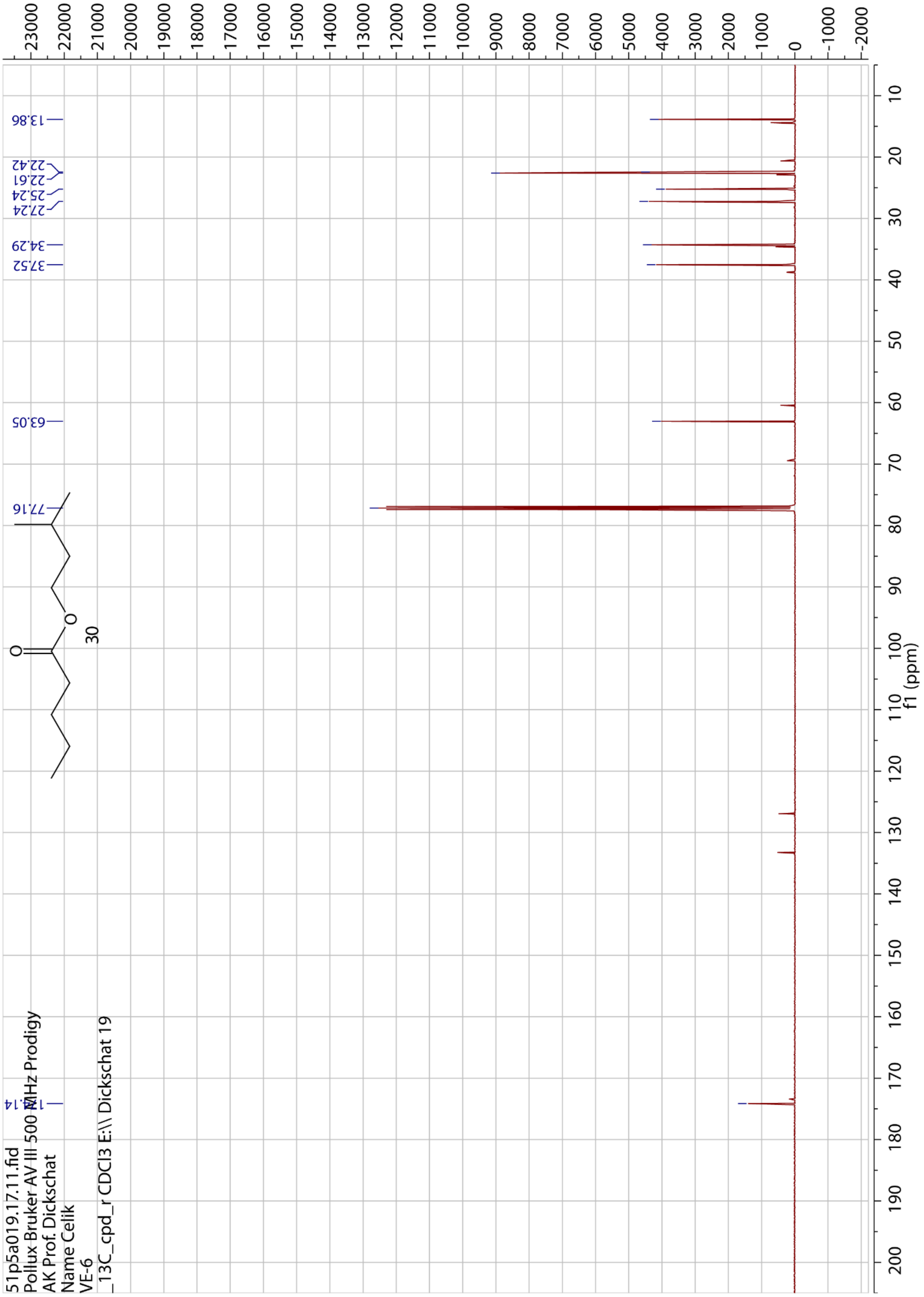

Figure S3: ^{13}C NMR spectrum of **30** (125 MHz, $CDCl_3$).

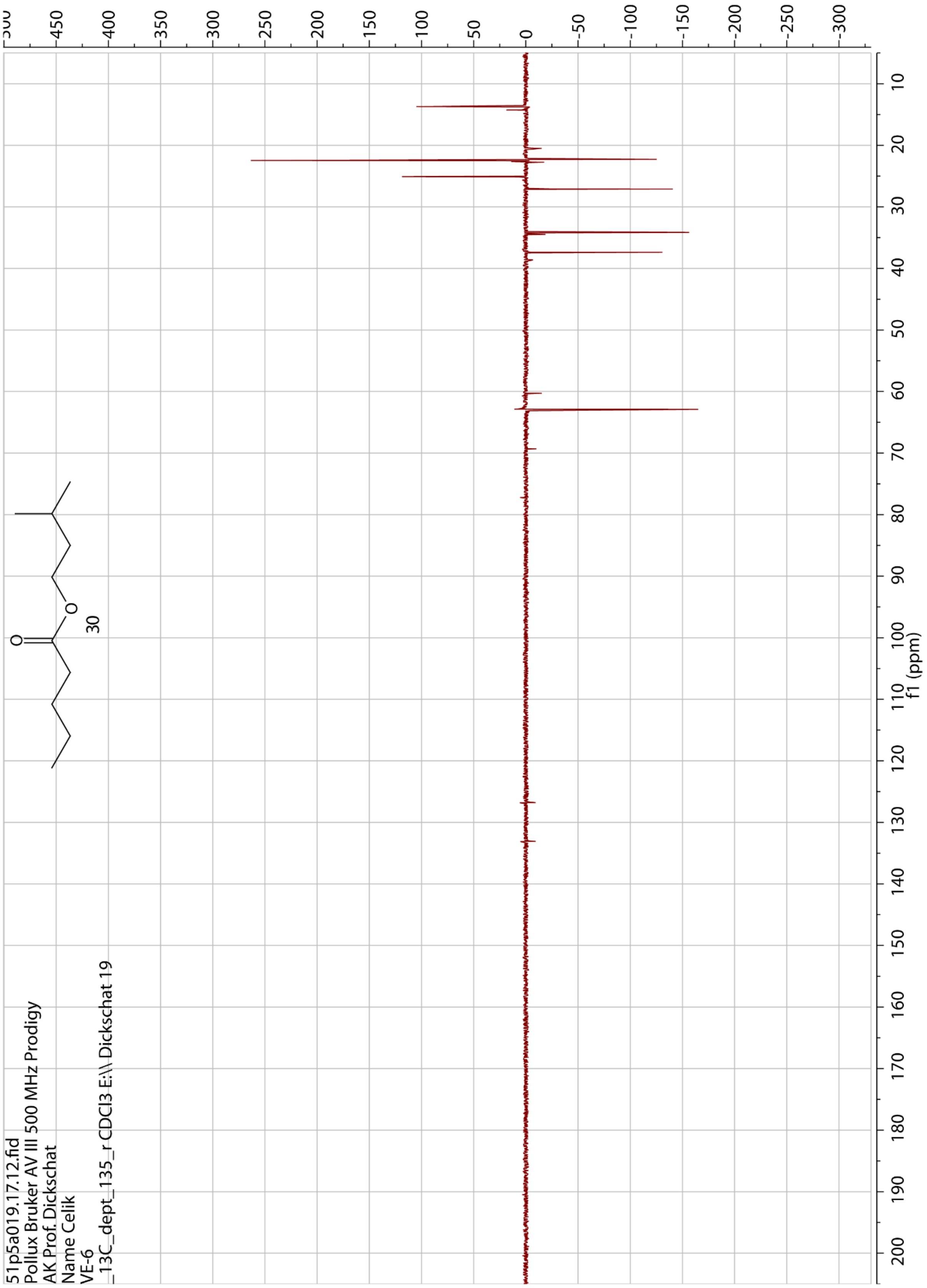

Figure S4: ^{13}C-DEPT spectrum of **30** (125 MHz, $CDCl_3$).

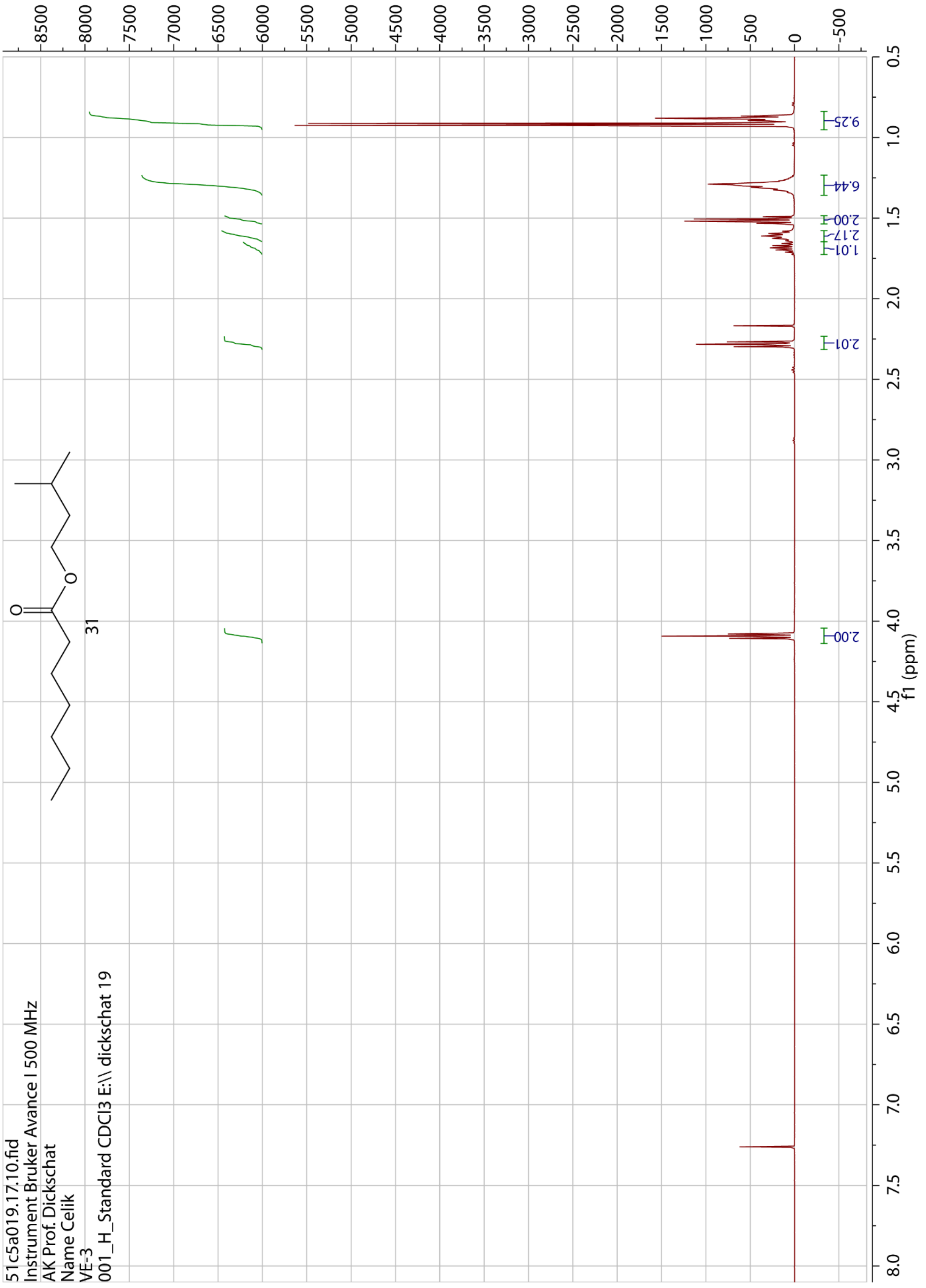

Figure S5: ^{1}H NMR spectrum of **31** (500 MHz, $CDCl_3$).

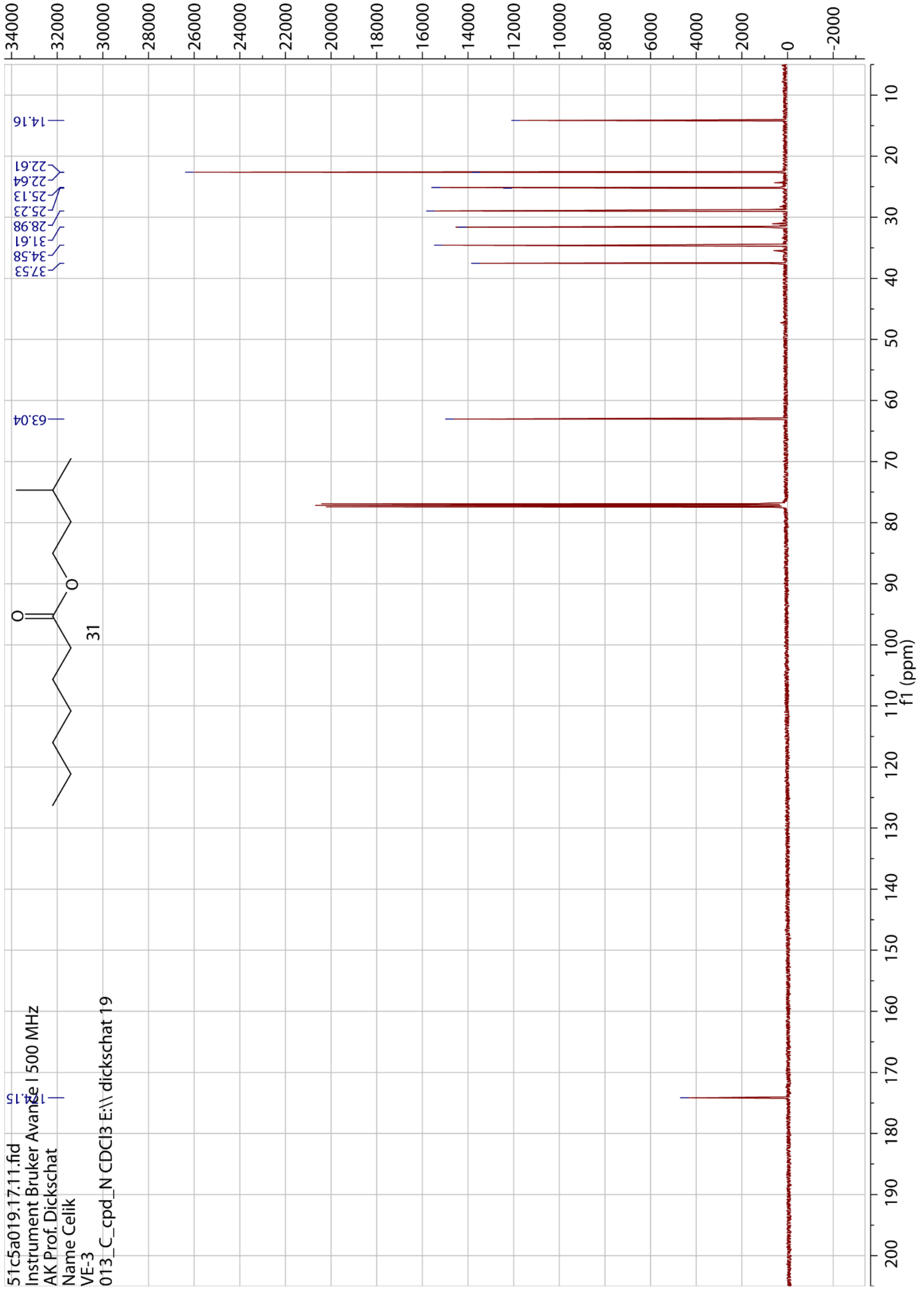

Figure S6: ^{13}C NMR spectrum of **31** (125 MHz, $CDCl_3$).

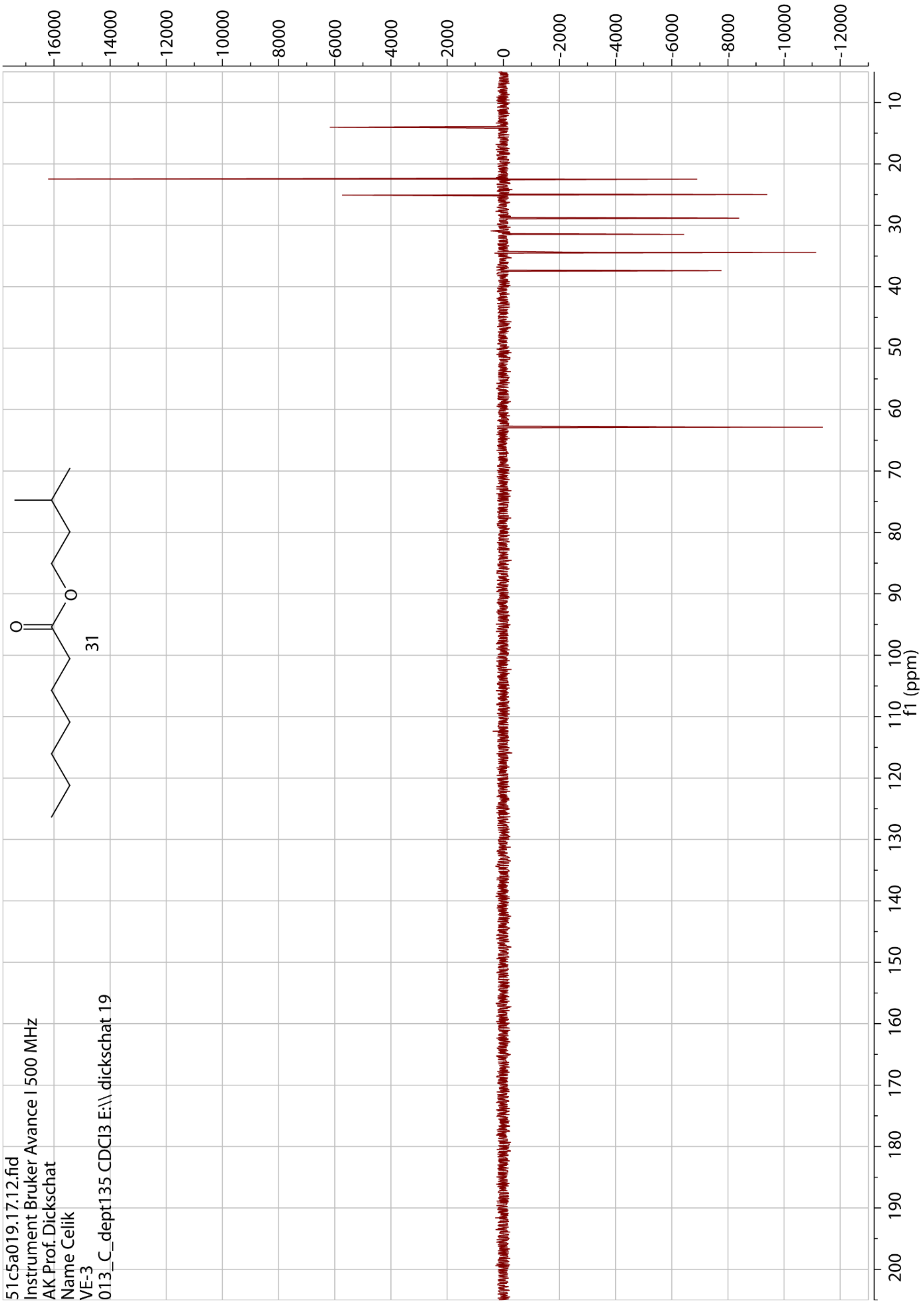

Figure S7: ^{13}C-DEPT spectrum of **31** (125 MHz, $CDCl_3$).

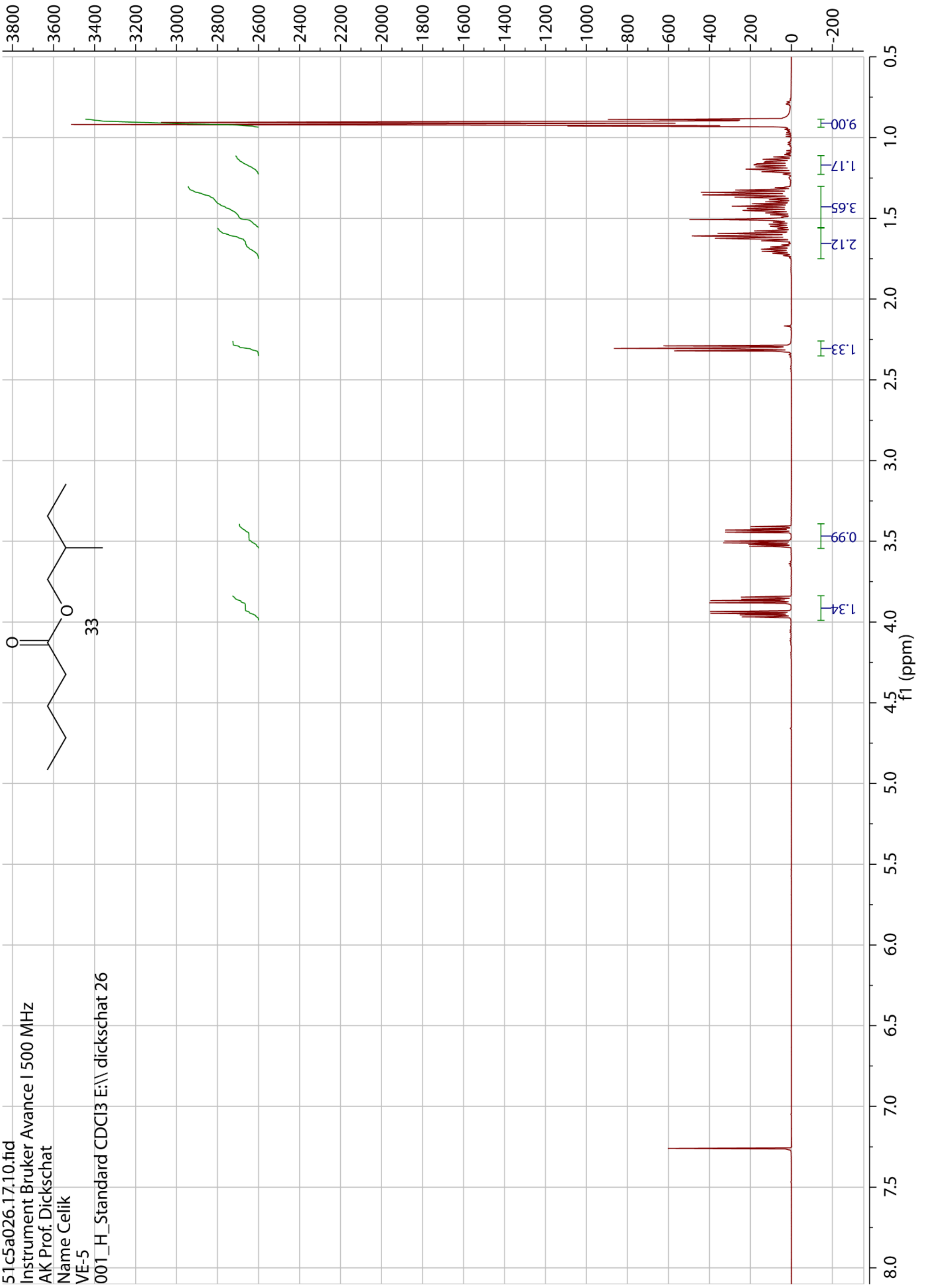

Figure S8: ^{1}H NMR spectrum of **33** (500 MHz, $CDCl_3$).

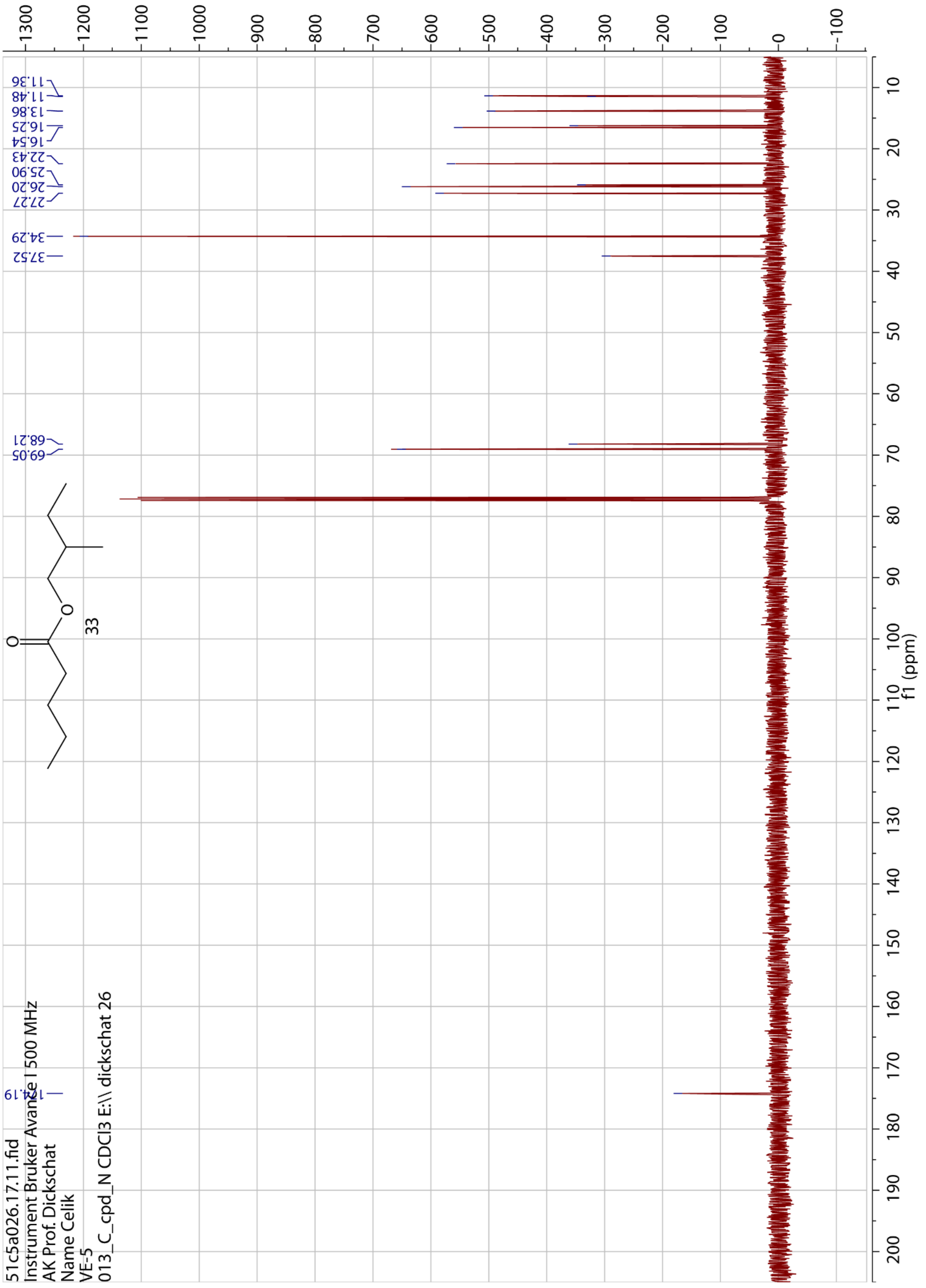

Figure S9: ^{13}C NMR spectrum of **33** (125 MHz, $CDCl_3$).

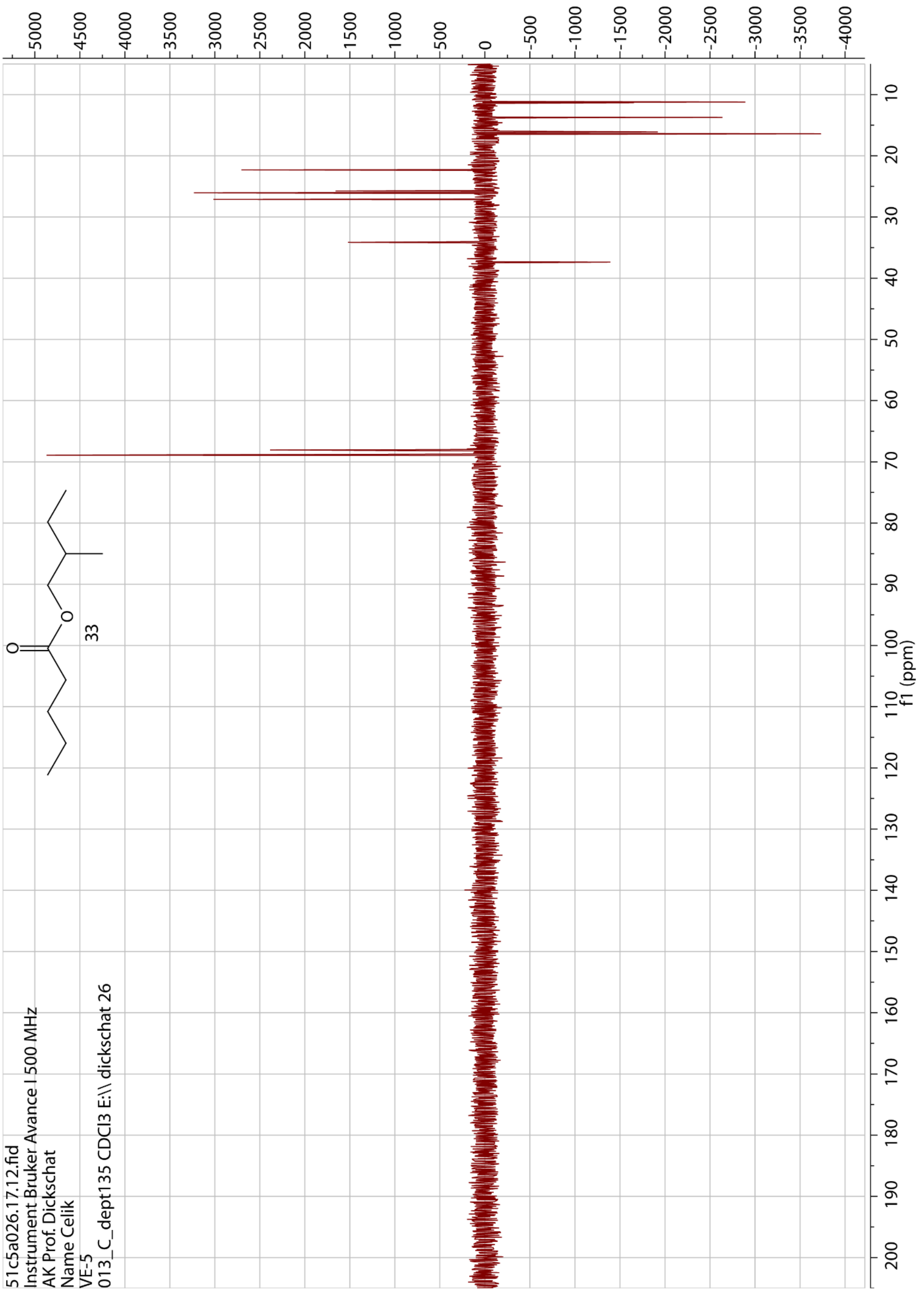

Figure S10: ^{13}C-DEPT spectrum of **33** (125 MHz, $CDCl_3$).

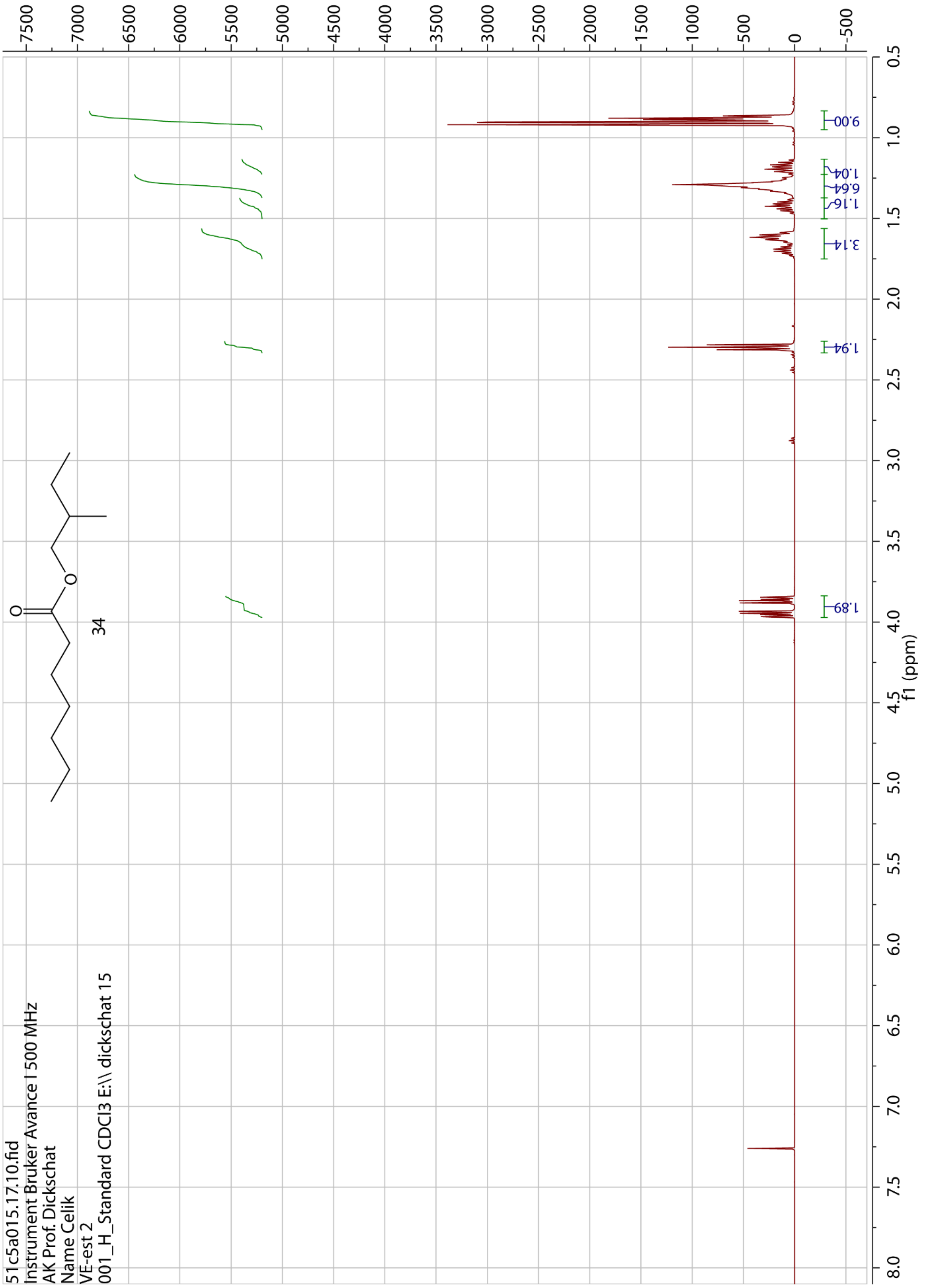

Figure S11: ^{1}H NMR spectrum of **34** (500 MHz, $CDCl_3$).

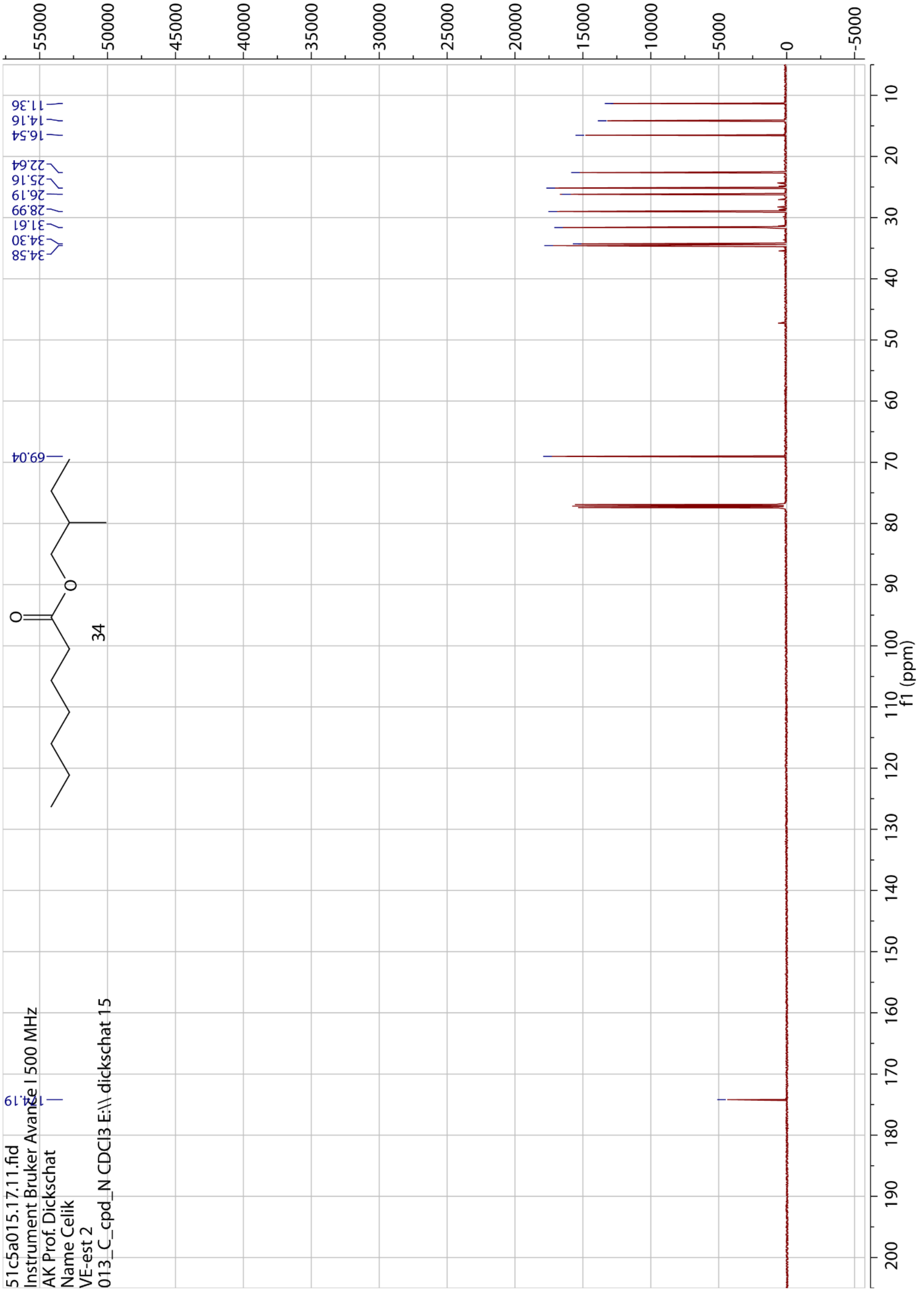

Figure S12: ^{13}C NMR spectrum of **34** (125 MHz, $CDCl_3$).

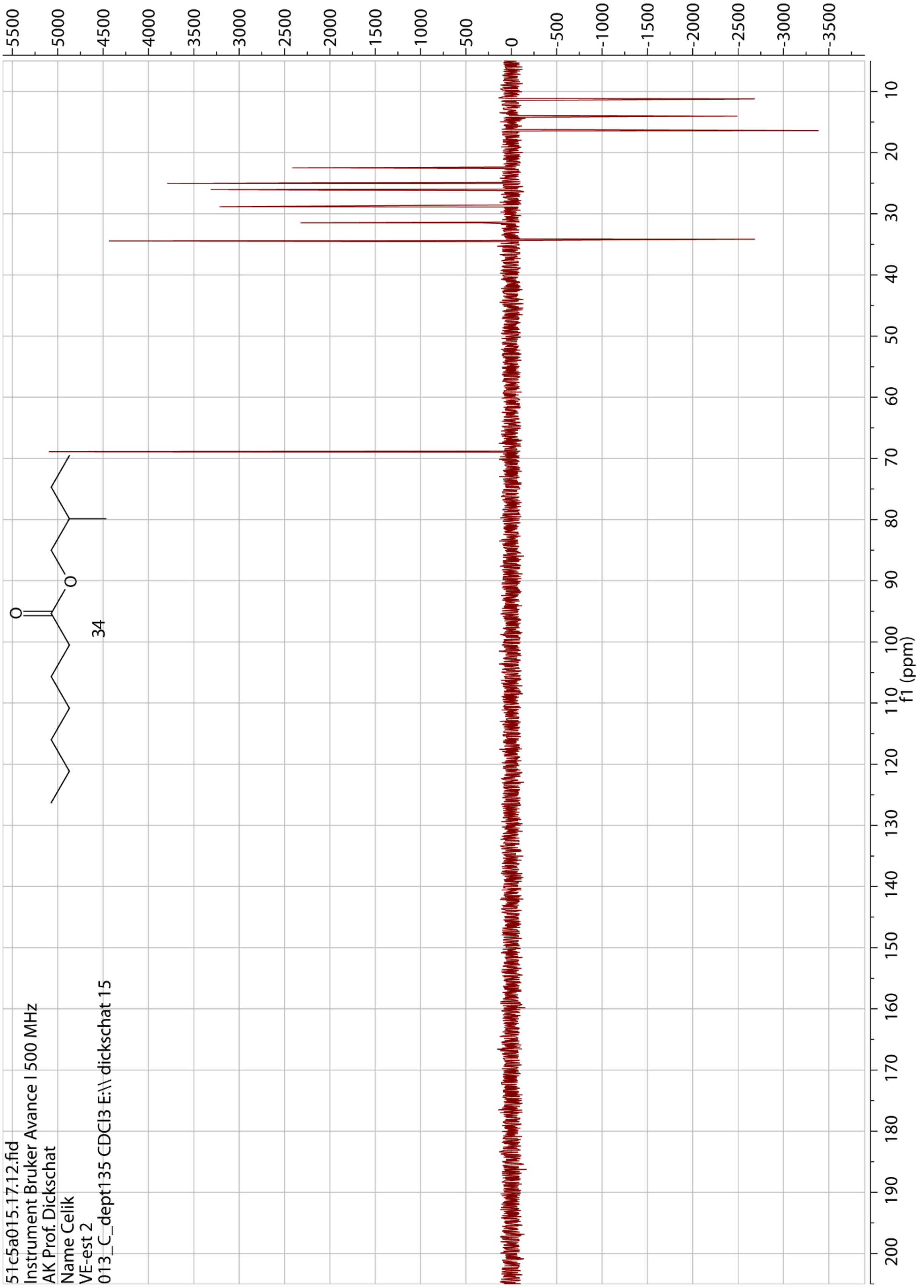

Figure S13: ^{13}C-DEPT spectrum of **34** (125 MHz, $CDCl_3$).

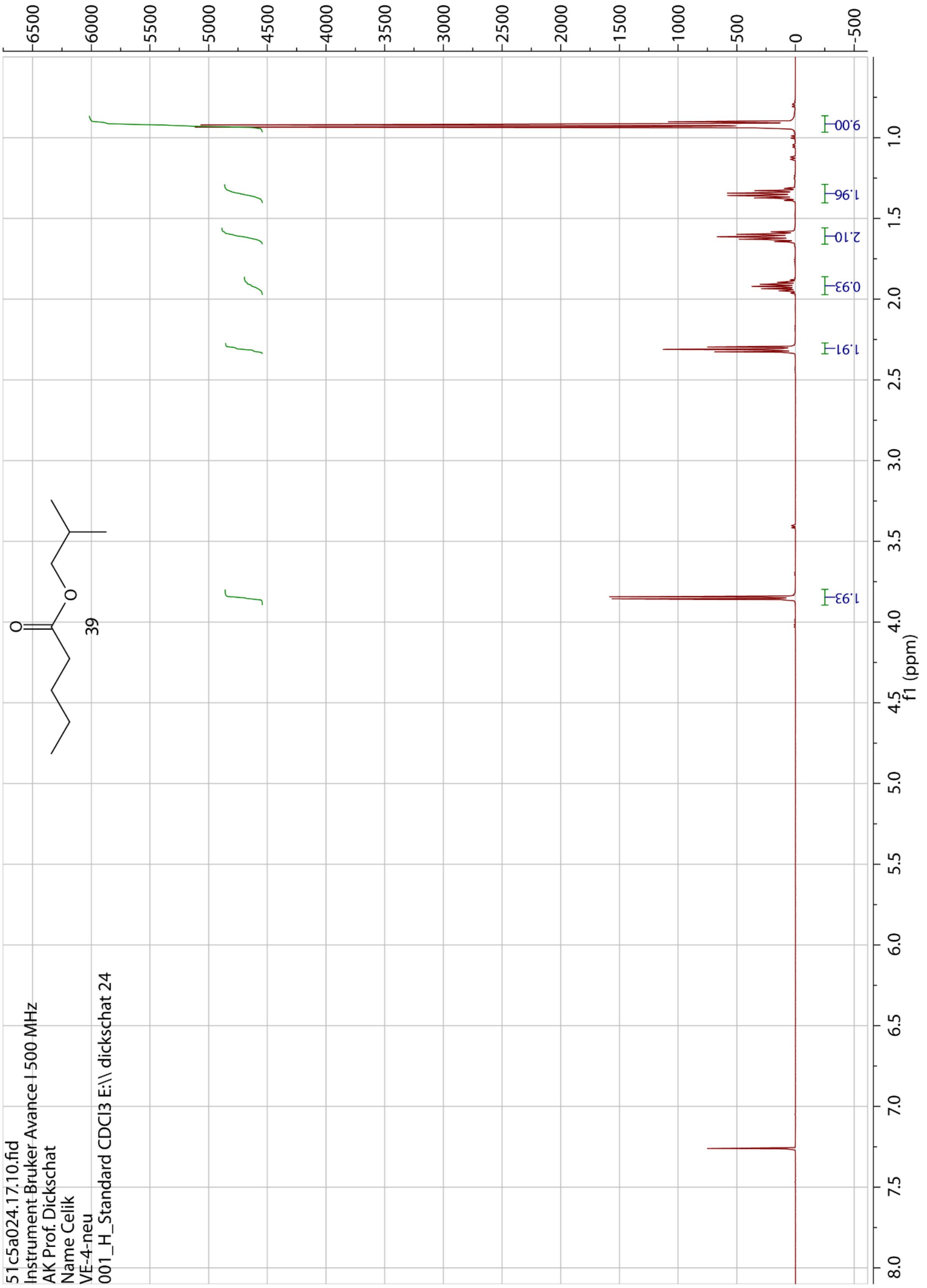

Figure S14: ^{1}H NMR spectrum of **39** (500 MHz, $CDCl_3$).

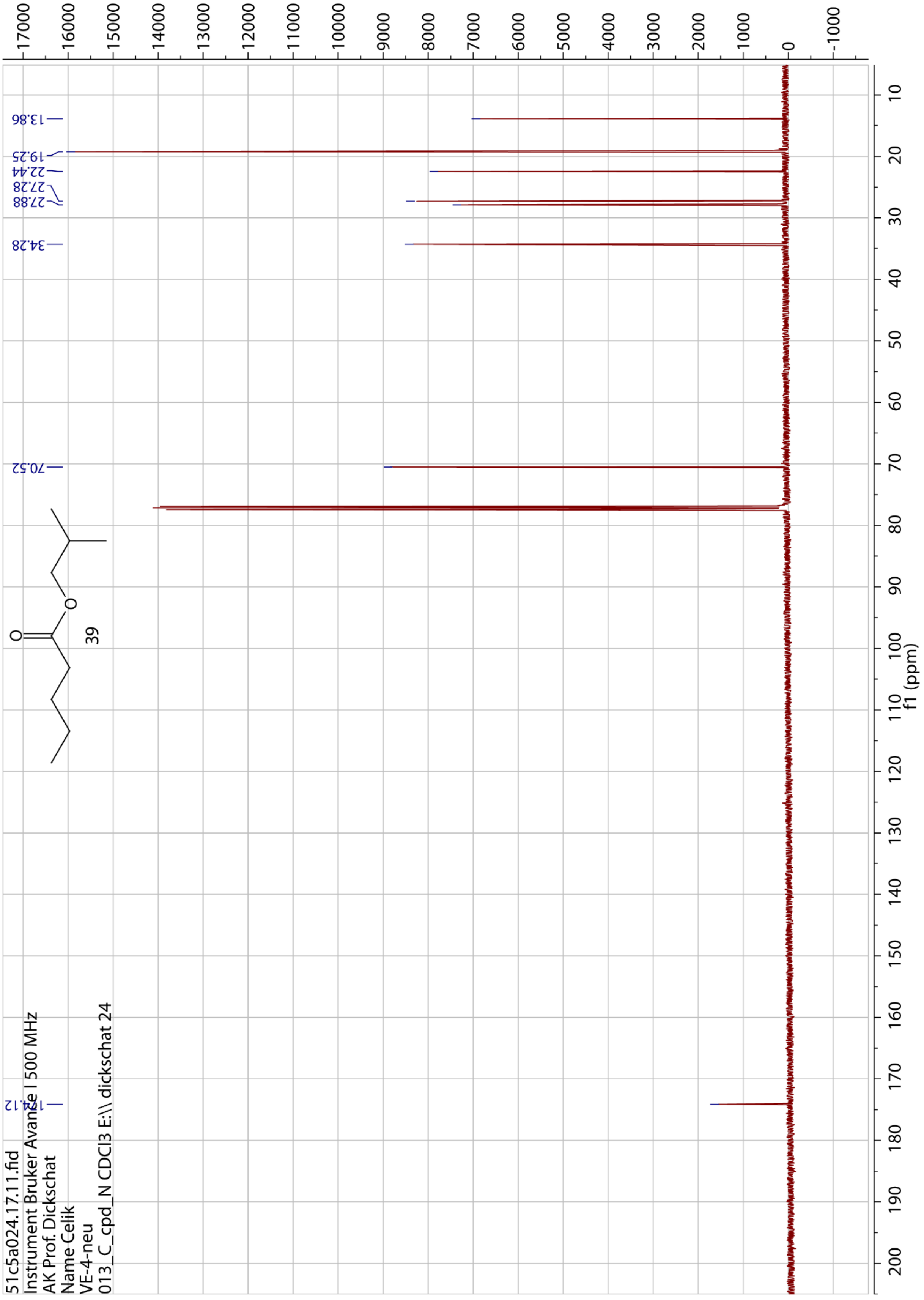

Figure S15: ^{13}C NMR spectrum of **39** (125 MHz, $CDCl_3$).

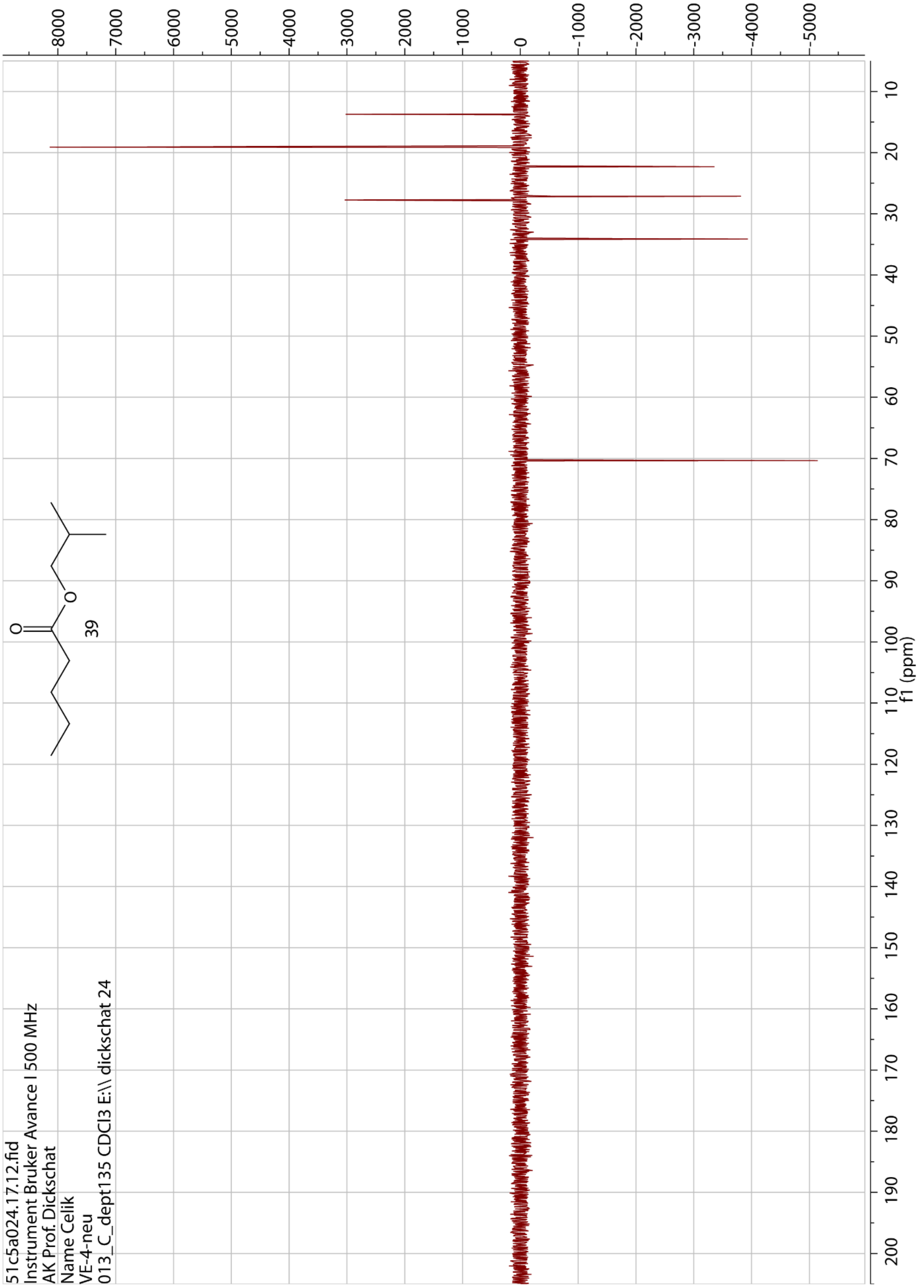

Figure S16: ^{13}C-DEPT spectrum of **39** (125 MHz, $CDCl_3$).

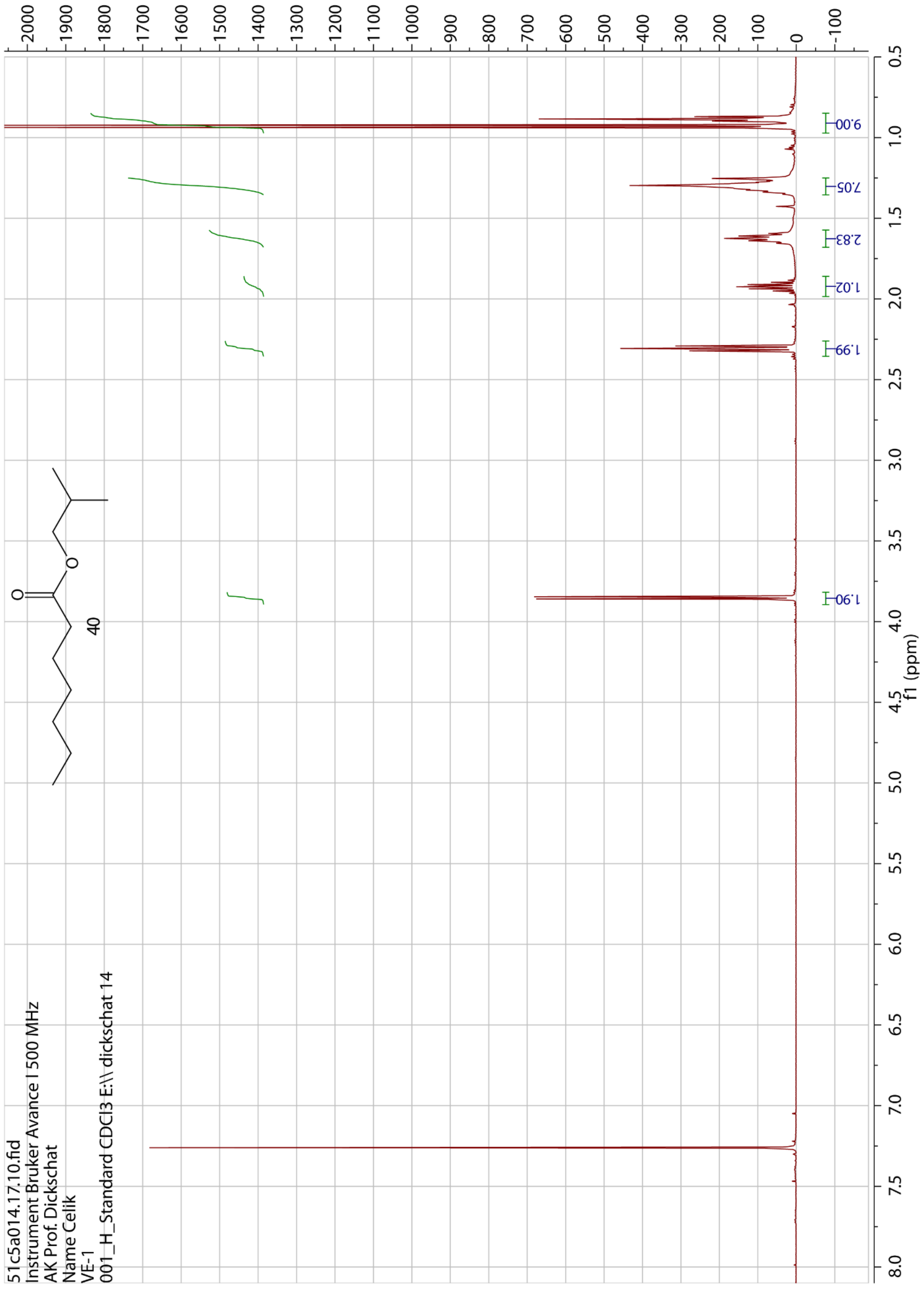

Figure S17: ^{1}H NMR spectrum of **40** (500 MHz, $CDCl_3$).

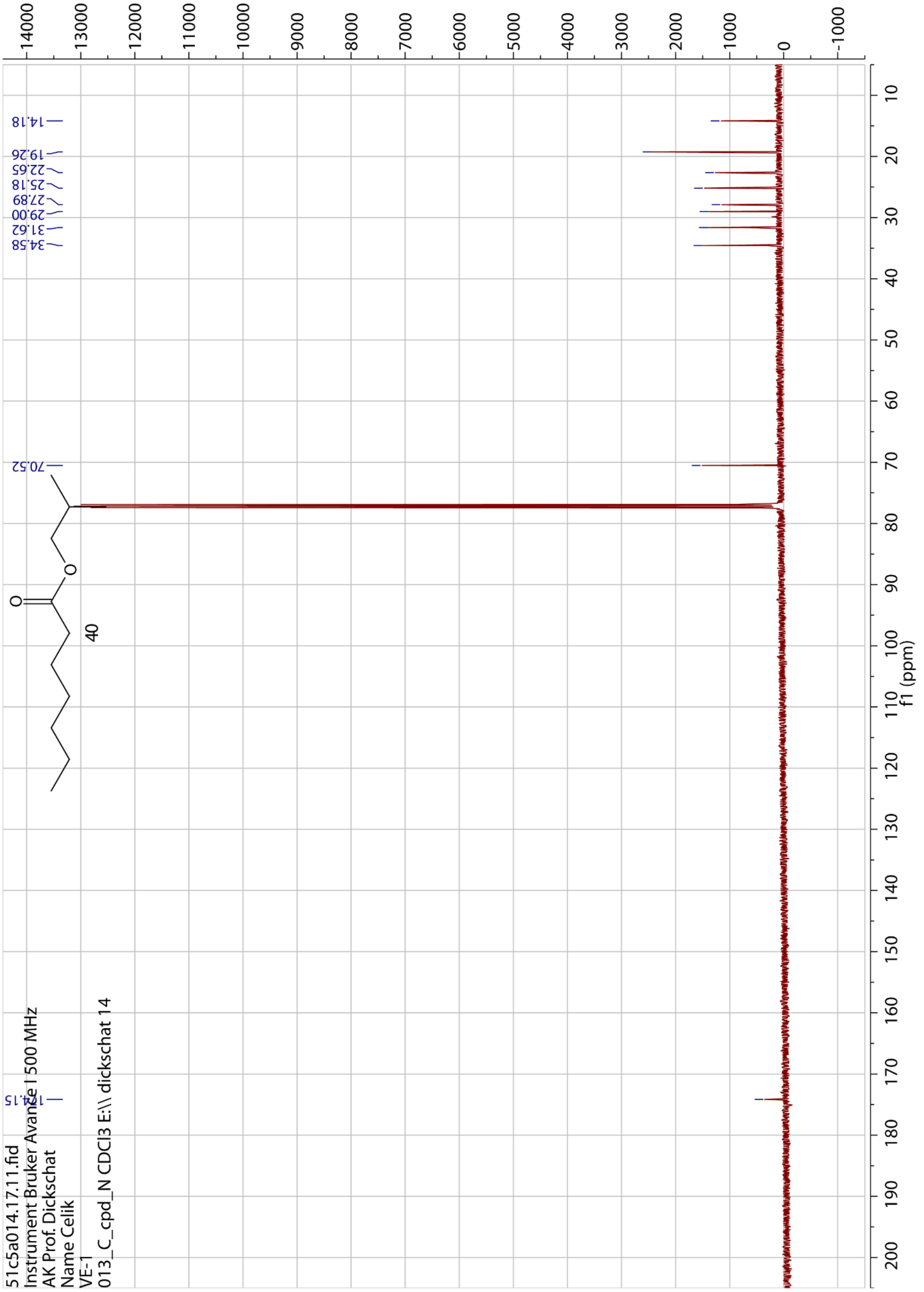

Figure S18: ^{13}C NMR spectrum of **40** (125 MHz, $CDCl_3$).

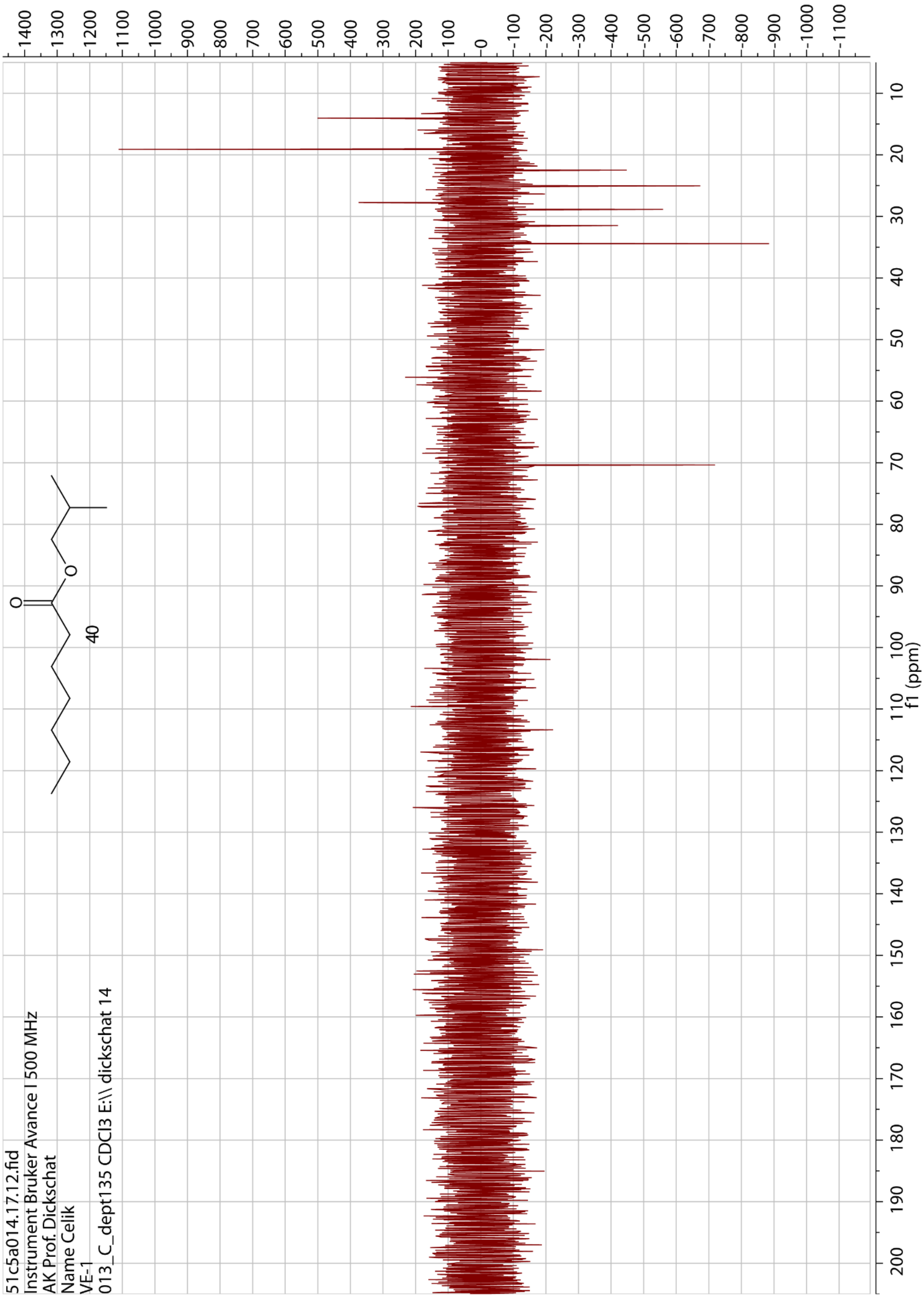

Figure S19: ^{13}C-DEPT spectrum of **40** (125 MHz, $CDCl_3$).

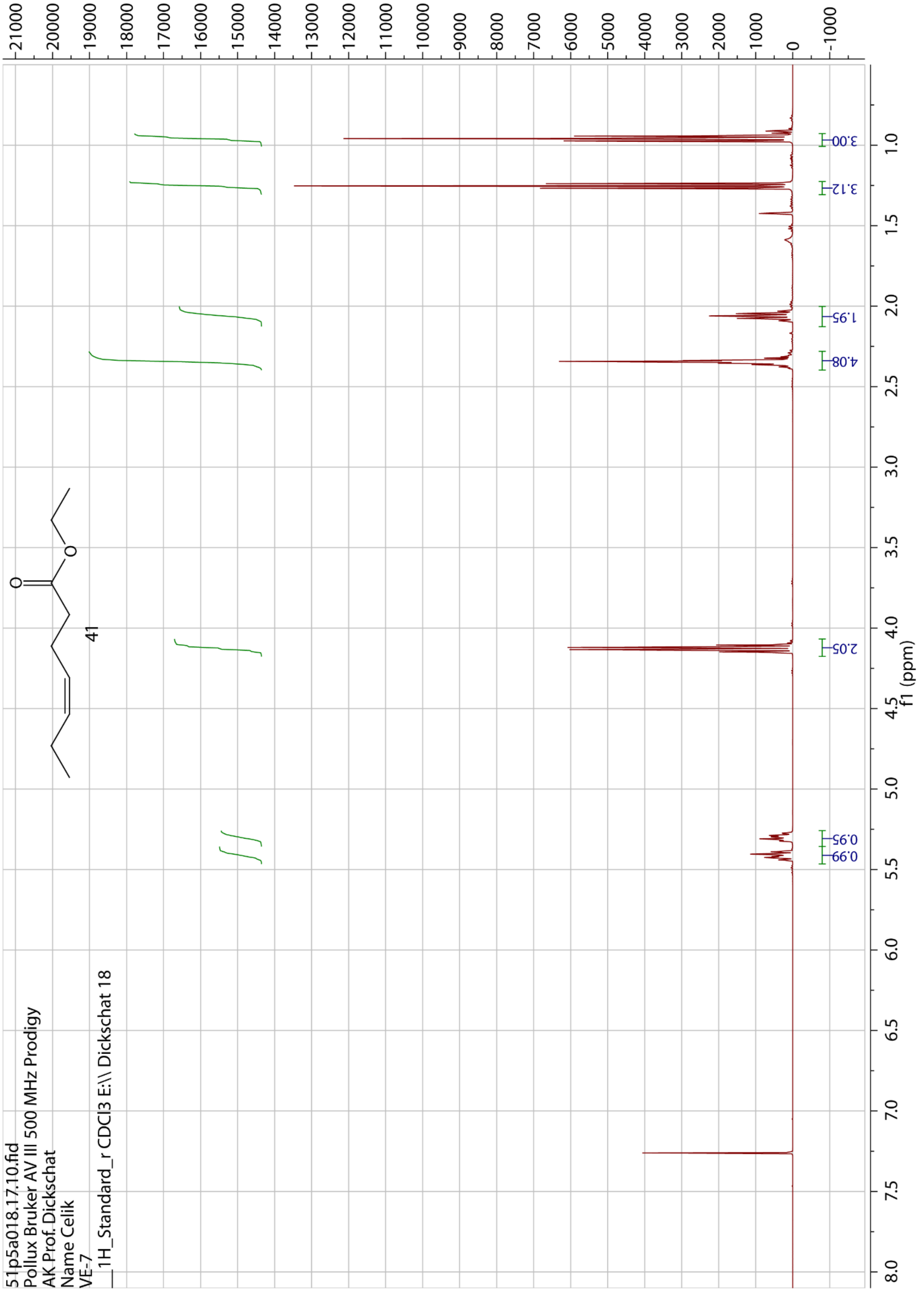

Figure S20: ^{1}H NMR spectrum of **41** (500 MHz, $CDCl_3$).

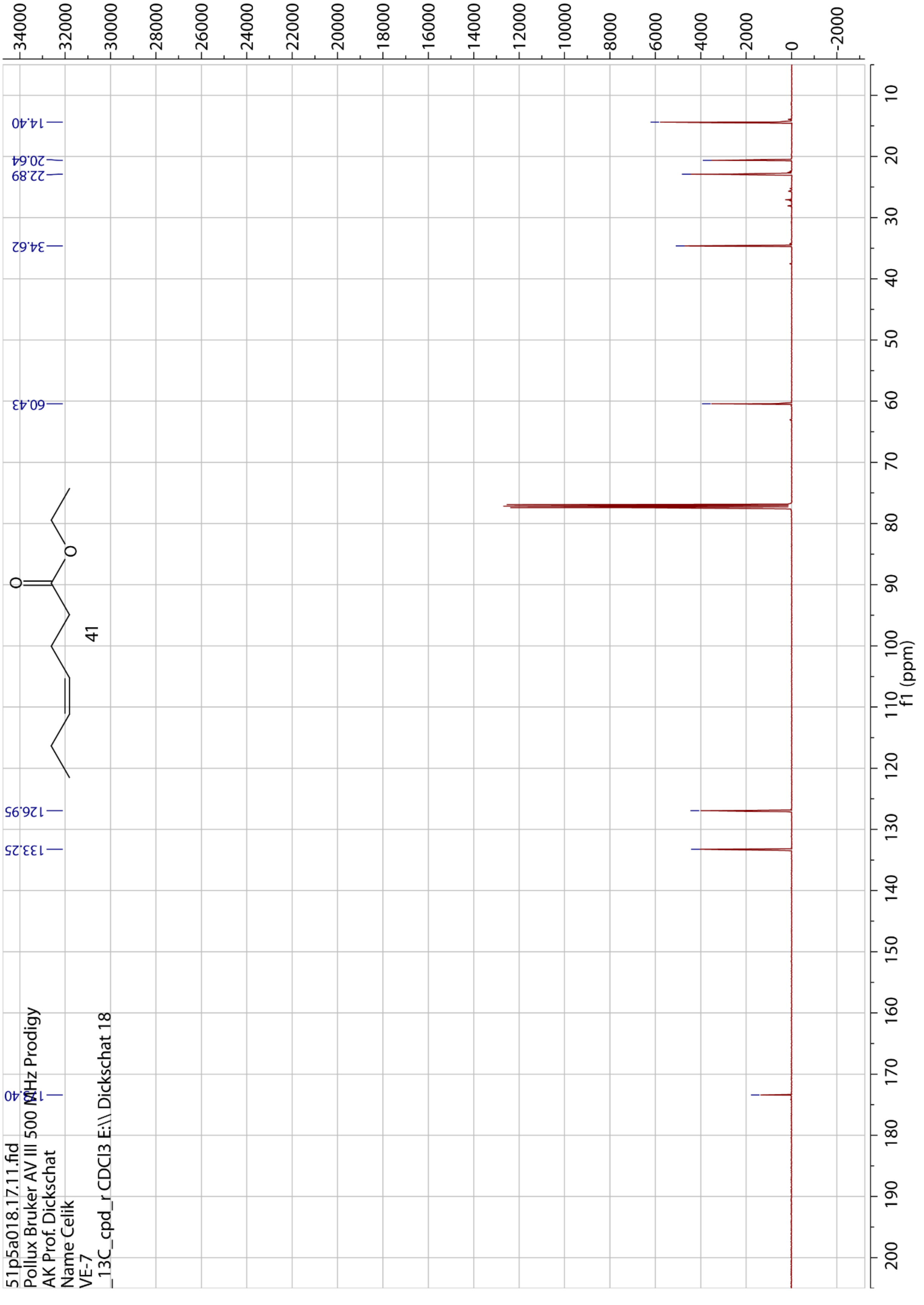

Figure S21: ^{13}C NMR spectrum of **41** (125 MHz, $CDCl_3$).

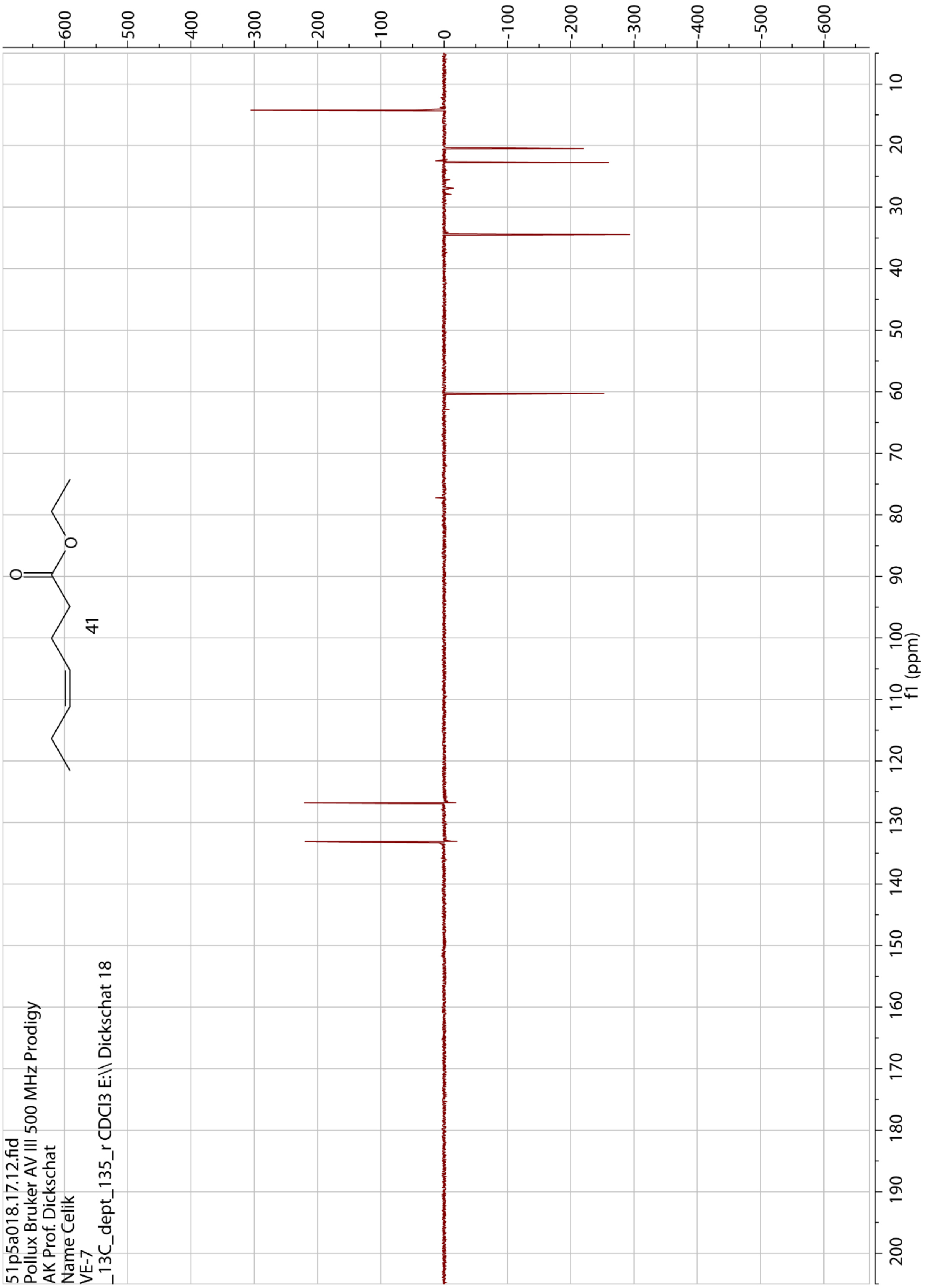

Figure S22: ^{13}C-DEPT spectrum of **41** (125 MHz, $CDCl_3$).
